# Latrophilins: A Neuro-Centric View of an Evolutionary Conserved Adhesion G Protein-Coupled Receptor Subfamily

**DOI:** 10.3389/fnins.2019.00700

**Published:** 2019-07-09

**Authors:** Ana L. Moreno-Salinas, Monserrat Avila-Zozaya, Paul Ugalde-Silva, David A. Hernández-Guzmán, Fanis Missirlis, Antony A. Boucard

**Affiliations:** ^1^Department of Cell Biology, Centro de Investigación y de Estudios Avanzados del Instituto Politécnico Nacional (CINVESTAV-IPN), Mexico City, Mexico; ^2^Department of Physiology, Biophysics and Neurosciences, Centro de Investigación y de Estudios Avanzados del Instituto Politécnico Nacional (CINVESTAV-IPN), Mexico City, Mexico

**Keywords:** latrophilin, teneurin, adhesion G protein-coupled receptors, cell adhesion molecules, neuronal synapse, alternative splicing, actin cytoskeleton, psychiatric disorders

## Abstract

The adhesion G protein-coupled receptors latrophilins have been in the limelight for more than 20 years since their discovery as calcium-independent receptors for α-latrotoxin, a spider venom toxin with potent activity directed at neurotransmitter release from a variety of synapse types. Latrophilins are highly expressed in the nervous system. Although a substantial amount of studies has been conducted to describe the role of latrophilins in the toxin-mediated action, the recent identification of endogenous ligands for these receptors helped confirm their function as mediators of adhesion events. Here we hypothesize a role for latrophilins in inter-neuronal contacts and the formation of neuronal networks and we review the most recent information on their role in neurons. We explore molecular, cellular and behavioral aspects related to latrophilin adhesion function in mice, zebrafish, *Drosophila melanogaster* and *Caenorhabditis elegans*, in physiological and pathophysiological conditions, including autism spectrum, bipolar, attention deficit and hyperactivity and substance use disorders.

## Latrophilins, 22 Years After Their Discovery

As the field of research on Adhesion G Protein-Coupled Receptors (aGPCR) is rapidly expanding, so is the interest for many of its subfamilies given their involvement in various physiological and pathophysiological events relevant to human health. A prototypical aGPCR subfamily named the latrophilins has attracted attention for more than 20 years since their discovery as part of an effort to identify the biological target mediating the calcium-independent effects of α-latrotoxin, a potent neurotoxin from the black widow venom (Krasnoperov et al., 1997; Lelianova et al., 1997; Sugita et al., 1998). Latrophilins qualify as prototypical because the study of these proteins provided many landmark discoveries that have later paved the way for understanding aGPCRs structure and function in general. Fast-forward 22 years later what do we know about latrophilins? Here, after reviewing many studies, we can only start formulating the broad realm of their function: the widespread expression of latrophilin receptors in many tissues uncovers just the tip of the iceberg; their role in neuronal tissues places latrophilins at the crown of prototypical aGPCRs.

## Latrophilin Domain Organization

**FIGURE 1:**
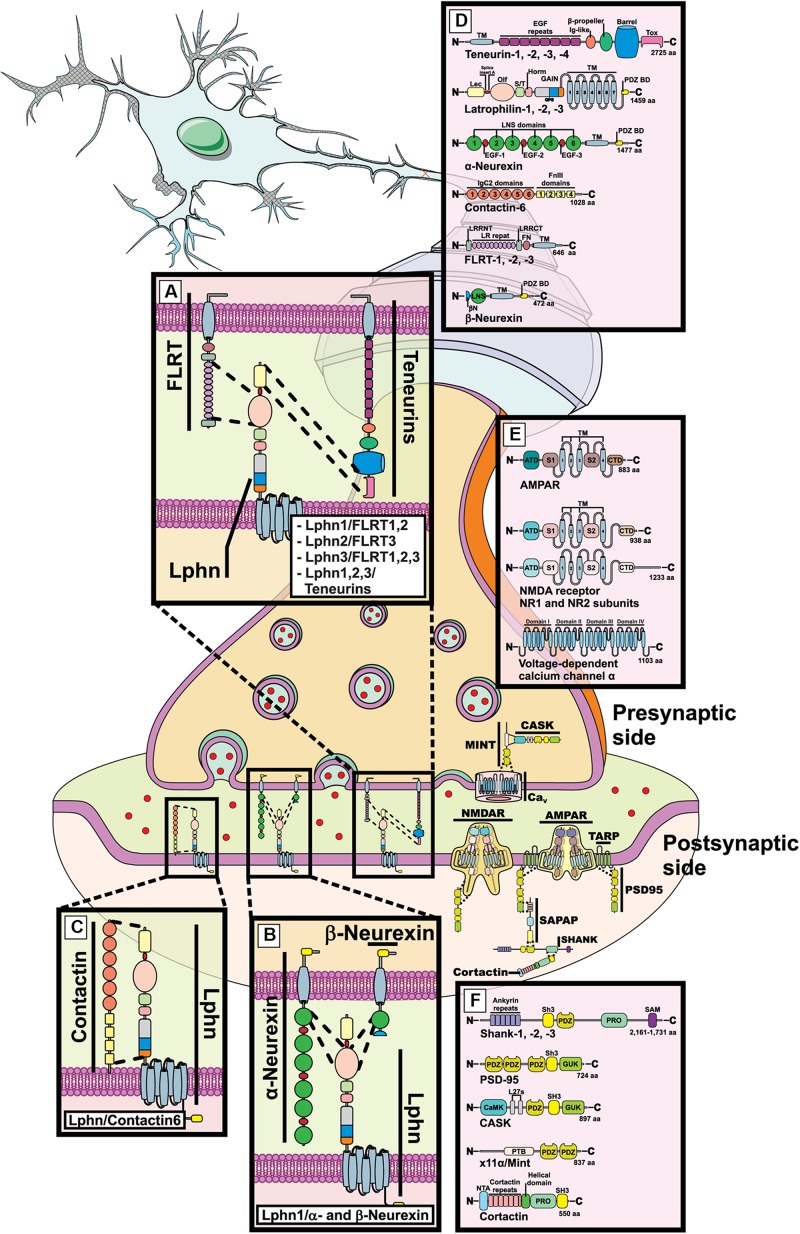
Latrophilin-ligands pairings at the mammalian synapse. Representation of a mammalian mature synaptic formation with pre- and post-synaptic compartments schematized. **(A–D)** Molecular complexes are shown between latrophilins and indicated ligands in dedicated zoomed-in boxes. **(E,F)** Components of excitatory synapses are shown such as N-methyl-D-aspartate receptor (NMDAR), α-amino-3-hydroxy-5-methyl-4-isoxazolepropionic acid receptor (AMPAR), PSD95, SHANK, Cortactin, MINT, CASK, voltage-dependent calcium channel α. **(D–F)** Indicated domains are the following: LNS, laminin, neurexin and sex-hormone binding; EGF, epidermal growth factor; TM, transmembrane; PDZ B.M., PSD95, Dlg, Zona occludens binding domain; Lec, Lectin; Olf, Olfactomedin; S/T, serine-threonine rich; Horm, hormone binding; GAIN, GPCR autoproteolysis inducing; Tox, Toxin; FN/FnIII, fibronectin type III; LRR/LR, Leucine-rich repeats; SH3, Src homology 3; GUK, guanylate kinase; CamK, Ca^2+^-Calmodulin kinase; PRO, proline rich; SAM, SH3 and multiple ankyrin repeat; PTB, phosphotyrosine binding; ATD, amino-terminal domain; VFT, Venus fly trap; CRD, cysteine rich domain.

Latrophilins are composed of the following domains which are schematized in Figure 1: two adhesion modules, the Lectin and Olfactomedin domains (the latter being absent in invertebrates); followed by a Hormone Binding Region adjacent to a GPCR autoproteolytic inducing domain (GAIN) which encompasses a cleavage site (GPS); and a GPCR region characterized by seven transmembrane helices with interconnecting loops and a C-terminal tail. The autoproteolytic event generates a bipartite protein composed of an extracellular N-terminal fragment (NTF) and a C-terminal fragment (CTF), with both fragments non-covalently linked to each other at the cell membrane (Arac et al., 2012). Latrophilins are among the most conserved aGPCRs with a presence that spans a wide spectrum of the evolutionary tree, suggesting that they may contribute to important functions in neuronal physiology (Krishnan et al., 2016).

## Endogenous Ligands for Latrophilins

Latrophilins are expressed as three isoforms in mammals (Krishnan et al., 2016) and are receptors for a variety of ligands. While some ligands appear to be common to all three isoforms, others are rather restricted to some isoforms. The list of ligands has been growing with the discovery of teneurin-2 (also known as Lasso, latrophilin associated protein; splice variant) (Silva et al., 2011), followed by neurexins (Boucard et al., 2012), FLRT (O’Sullivan et al., 2012) and finally contactin-6 (Zuko et al., 2016).

### Teneurins

The first family of endogenously expressed extracellular ligands described for latrophilins comprise members of a four-isoforms group in mammals: teneurin-1, -2, -3, and -4 (Silva et al., 2011). Out of these high molecular weight proteins, teneurin-2 or Lasso (teneurin-2 splice variant), was the first to be identified as a high-affinity partner for latrophilins although the remaining isoforms were subsequently included as part of the potential interactors (Silva et al., 2011; O’Sullivan et al., 2012, 2014; Boucard et al., 2014). As type-II membrane proteins teneurins project their c-terminal adhesion domains toward the extracellular media to consolidate their interaction with latrophilins (Figure 1A). Such interaction mainly occurs between the extreme c-terminal region of teneurin and the Lectin-like domain of latrophilins but requires the additional contribution of the Olfactomedin domain in order to reconstitute a high-affinity binding site (Figure 1A; Boucard et al., 2014). As is the case for other adhesion molecule families, alternative splicing modifies the quaternary structure of teneurins, a mechanism that has recently been reported to generate homophilic adhesion complexes stabilizing cell-cell contacts (Berns et al., 2018). The teneurin-latrophilin pair has also been the first fully functional complex to be characterized, as their interaction not only stabilizes intercellular adhesion but also generates an intracellular signal involved in modulating calcium levels and/or cAMP related pathways (Muller et al., 2015; Li et al., 2018; Vysokov et al., 2018). It is noteworthy that in addition to the presence of alternative splicing, teneurin proteins can also generate c-terminally cleaved products known as teneurin C-terminal Associated Proteins or TCAP, which are capable of regulating events as diverse as metabolism and reproduction but also neuronal morphology (Al Chawaf et al., 2007; Colacci et al., 2015; Hogg et al., 2018). The evidence that TCAP sequences overlap with the proposed latrophilin binding-domain makes them likely candidates as latrophilin ligands and recent studies suggest that TCAP-mediated effects require a functional interaction with latrophilins (Silva et al., 2011; Husic et al., 2019).

### Neurexins

This family of type I proteins is expressed by three genes in mammals, each producing two main isoforms: the large isoforms, α-neurexins, and the short isoforms known as β-neurexins (Figure 1B). As a consequence of extensive alternative splicing, these molecules present a highly polymorphic profile with the potential to interact with different sets of partners/ligands (Treutlein et al., 2014). The binding of neurexins to latrophilins is strictly regulated by alternative splicing of the former (Boucard et al., 2012). However, despite the fact that all three latrophilin isoforms possess the highly homologous Olfactomedin domain, only latrophilin-1 was shown to establish heterophilic contact with neurexins through that domain to stabilize intercellular adhesion while attempts to demonstrate similar binding for latrophilin-2 and latrophilin-3 have failed (Figure 1B; Boucard et al., 2012; O’Sullivan et al., 2014; Zuko et al., 2016). Interestingly, both neurexins and latrophilins have been described as neuronal receptors for α-latrotoxin, a potent component of the black widow spider venom which acts on the presynaptic compartment in order to induce massive neurotransmitter release. In particular, neurexin-1α and latrophilin-1 were thought to account for the majority of binding sites targeted by the neurotoxin in neuronal tissues (Tobaben et al., 2002). It is still unclear how contact between both latrophilin and neurexin leads to neuronal synapse formation but their genetic interdependence in mice brains suggests a yet unknown functional mechanism that warrants further investigation (Tobaben et al., 2002).

### FLRT

Previously known for their role in cell migration, the Fibronectin and Leucine-Rich Transmembrane proteins or FLRT were identified as high-affinity ligands for latrophilins in brain tissues (O’Sullivan et al., 2012). Ubiquitously expressed as 3 isoforms in vertebrates (FLRT1, 2, and 3), most of FLRT functions in neurons have been attributed to adhesion events mediated by homophilic contacts or repulsion events through their heterophilic interaction with Unc5 family of membrane receptors that respond to guidance cues (Yamagishi et al., 2011; Seiradake et al., 2014). Thus, the Unc5-FLRT pair forms a chemorepellent complex while FLRT-FLRT interactions recapitulate an adhesive complex (Karaulanov et al., 2006; Seiradake et al., 2014). However, FLRT-mediated adhesion would prove to not only rely on homophilic binding but also on heterophilic interactions that came into light after latrophilins were identified as potential partners for FLRT (Figure 1A; O’Sullivan et al., 2012). Further characterization of FLRT structure would provide unexpected findings on this newly described interaction. The characterization of both Unc5-FLRT and FLRT-FLRT binding determinants denoted that FLRT leucine-rich repeats constituted the only domain necessary for maintaining the interaction and stabilizing intercellular adhesion or repulsion (Figure 1A; Karaulanov et al., 2006; Seiradake et al., 2014). Along the same line, the determinants establishing latrophilin-FLRT interaction were circumscribed by the exact same region which could potentially create a competitive interaction pattern between latrophilin, FLRT and Unc5, a situation that would allow the segregation between two contrasting functions of FLRT because repulsive (FLRT-Unc5) and adhesive (FLRT-latrophilin) functions would compete in order to be mutually exclusive as one would expect (Jackson et al., 2015, 2016; Lu et al., 2015). However, this scenario received counter-evidences following the elucidation of the latrophilin-FLRT-Unc5 crystal structure (Lu et al., 2015; Jackson et al., 2016). Indeed, the three molecules were observed as forming part of the same molecular complex facilitated by determinants of the “arc shaped” FLRT leucine-rich repeats (LRR) region: latrophilin interacted with LRR convex side while Unc5 interacted with LRR concave side, which is consistent with both molecules maintaining a non-competitive binding dynamic due to non-overlapping binding interfaces (Lu et al., 2015; Jackson et al., 2016). In the future, it will be interesting to elucidate which cellular functions are supported by the formation of these super-complexes and how these interactions are regulated in synaptogenesis events.

### Contactins

As part of the immunoglobulin cell adhesion molecules, contactins extracellular region consists of immunoglobulin-like and fibronectin repeats. Although contactins lack a transmembrane domain, they are linked to the cell surface via a GPI anchor, a feature that allows them to restrict their cellular localization to the cell membrane but that impedes them from autonomously initiating intracellular signaling. Contactins have been shown to form molecular complexes with various transmembrane proteins therefore allowing them to provide a complement to their signaling function due to the ability of the newly formed complexes to interact with cytoplasmic signaling cascades. Out of the six isoforms of contactins found in vertebrates (contactin-1,-2,-3,-4,-5, and -6), only contactin-6 was identified as a latrophilin ligand (Figure 1C). In contrast to previously described ligands, contactin-6 was unable to mediate *trans*-cellular adhesion through its contact with latrophilin-1 (Zuko et al., 2016). Instead of a contact in *trans* (between two separate cell membranes) a *cis*-configuration was the preferred description for this molecule pairing at the cell membrane. Indeed, not only were contactin-6 and latrophilin-1 expressed in the same neuronal cells (cortical neurons) but the effect of the molecular complex on apoptosis pathways and neuronal morphology was only appreciable in a cell-autonomous fashion (Zuko et al., 2016). While the protein domains involved in the stabilization of the contactin-6/latrophilin-1 complex are unknown, the question regarding their function in the adhesive properties of the cell remains open.

## Latrophilins: Establishing Neuronal Connections

### Growth Cones Formation

**FIGURE 2:**
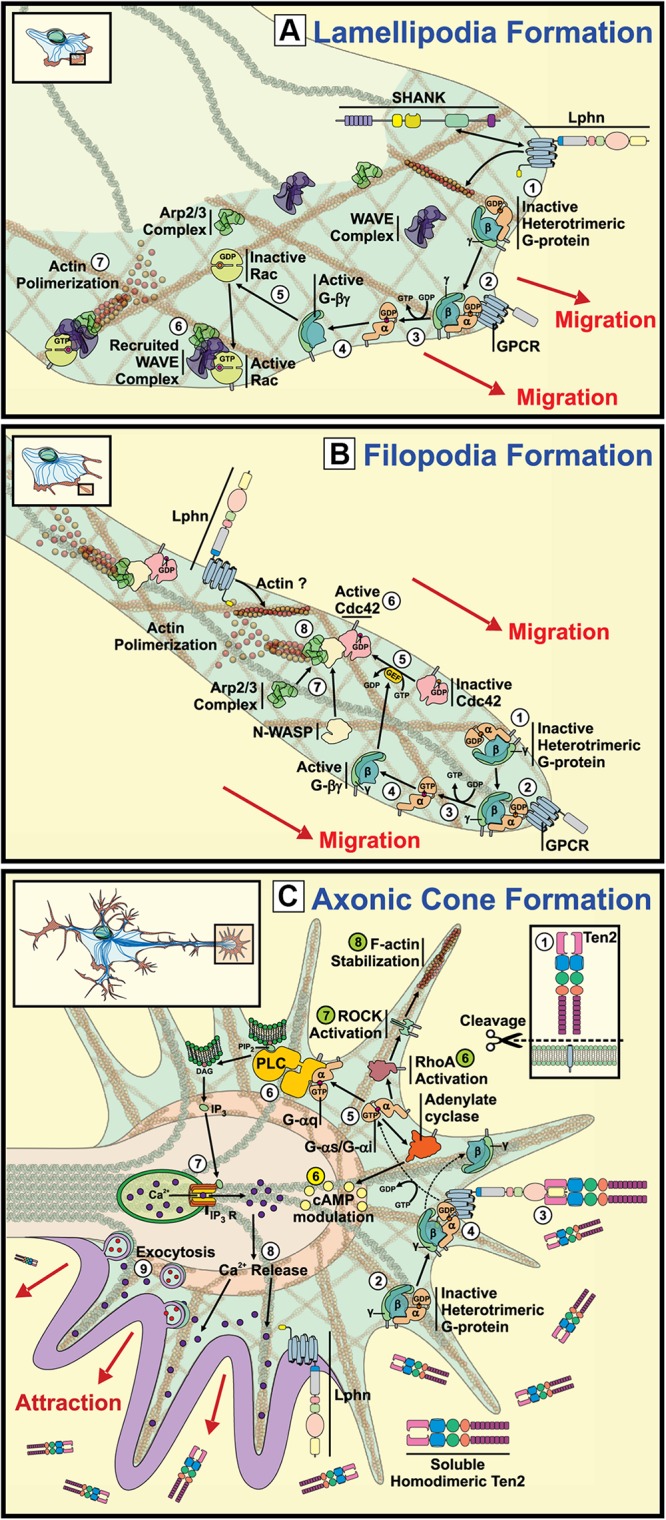
Signaling pathways underlying the potential involvement of latrophilins in growth cone and actin structures formation. Representation of growth cone structures and associated signaling. **(A)** *Lamellipodia Formation*: GPCR activation leads to their coupling with G proteins
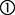

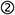
 provoking subunits dissociation
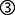

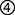
. Activation of the Rac pathway by Gβγ subunits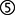 results in the recruitment of WAVE and ARP2/3 complexes at the front of migration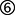 and generates actin polymerization (Lowery and Van Vactor, 2009; Chia et al., 2013). The reported interaction between Lphn1 and SHANK could presumptively couple the aGPCR to the actin cytoskeleton (Tobaben et al., 2000). **(B)** *Filipodia Formation*: Activation of G proteins by GPCR stimulation
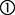

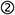
 at the migration front leads to dissociation of α subunits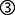 from βγ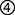 subunits, which will in turn activate small GTPase Cdc42
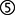

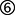
 recruiting n-WASP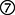 and ARP2/3 complexes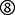 and provoking actin polymerization (Lowery and Van Vactor, 2009). **(C)** *Axonic Cone Formation*: Proteolytically cleaved teneurin-2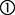 activates latrophilin
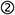

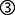

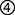
 leading to Gα_*q*_ protein induction of PLC
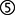

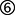
 followed by an increase in Ca^2+^ release from the endoplasmic reticulum through IP_3_ receptors
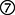

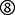
 (Vysokov et al., 2018). Alternatively, cAMP levels can be modulated by activation of Gαi or Gαs proteins 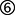
(yellow) (Muller et al., 2015; Nazarko et al., 2018). In parallel, G protein activation can also lead to the stimulation RhoA/ROCK pathway supporting filopodial formation through actin stabilization
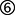

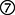

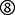
 (green) (Siehler, 2009).

In a developing neuronal network, the purpose of neuronal migration is presumably to help find the adequate partnering cell. This migration is facilitated by the elongation of axonal structures driven by extending microtubules onto which actin structures contribute to increasing the surface contact with the surroundings (Figures 2A–C). Such neuronal specializations that are represented by the formation of growth cones provide polarity and directionality to this active exploration/migration process (Rich and Terman, 2018). The dynamic nature of growth cones and their ability to respond quickly to ever changing environmental cues allows for a highly accurate target recognition process to occur, thus leading to precise interneuronal contacts. Molecular determinants that guide the formation and migration of growth cones have been identified, thus providing the initial description of how environmental cues can instruct migration patterns by engaging the cytoskeleton to induce movement (Lowery and Van Vactor, 2009). Among such molecules, the Ephrins and their receptors Ephs are probably the best described. A complex network of membrane-attached Ephrins and Ephs establish a chemical gradient throughout which neurons appendices will physically progress until the right target is found (Xu and Henkemeyer, 2012). Thus, the notion that migration cues should establish a molecular gradient in order for the neuronal protrusion to follow its course has permeated our knowledge of the molecular basis of growth cones formation and function. However, the vast diversity of neuron types suggests the existence of an equally diverse set of guidance cues and receptors.

Proteins that assist the formation of growth cones have been described using proteomic assays aimed at detecting proteins that are differentially distributed along growth cones versus the ones present in axons. Such assays revealed an enrichment of latrophilin-3 (Lphn3) at the tip of migration. Concurrently, neurexin1 followed the same expression pattern as for Lphn3, whereas teneurin-2 was equally distributed along both structures (Nozumi et al., 2009). Enrichment of Lphn3 at these growth cones was accompanied by actin remodeling proteins such as cofilin, and proteins from neurotransmitter vesicles release machinery such as Munc18, Snap25 or Synaptotagmin, thus reinforcing the subcellular localization of latrophilins as presynaptic proteins.

Cementing the role of latrophilin in growth cone migration, a study conducted by Vysokov et al. (2018) evidenced the importance of the right Lphn/ligand pairing for providing the instructional signals to achieve axonal elongation and directionality (Figure 2C; Vysokov et al., 2018). Indeed, the group showed that hippocampal neurons responded to a gradient of a secreted splice variant of teneurin-2 by sending a higher number of axons toward the established gradient than in control conditions not exposed to soluble teneurin-2. This effect was greatly dependent on Lphn1 expression as Lphn1-deficient neurons failed to respond to such teneurin-2 gradient, thus suggesting that latrophilin and teneurin heterophilic contacts support growth cone formation (Vysokov et al., 2018). In support of these observations, a functional teneurin-1 deficiency in *C. elegans* revealed neuronal pathfinding defects in pharyngeal neurons development, a process that seems to be engaging components of both the extracellular matrix and of the actin cytoskeleton which constitute important elements of axon-guidance events (Drabikowski et al., 2005; Morck et al., 2010). However, studies of hippocampal neurons from teneurin-3 deficient mice have provided contrasting evidences that rather point to the importance of a splicing-dependent homophilic teneurin-teneurin contact in instructing neuronal wiring (Berns et al., 2018). It will be interesting to witness how these paradigms will be reconciled in the future as they might reveal unknown mechanisms of action for this cooperating pair of adhesion molecules.

#### Latrophilins and Modulation of Actin Cytoskeleton Elements

Whether cell migration events require Lphn/teneurin or teneurin/teneurin interactions, evidences highlight the possible involvement of these molecules in reshaping the cell cytoskeleton. This remodeling is essential for allowing the formation or retraction of contact structures such as filopodia and lamellipodia, actin-rich protrusions that increase the surface contact with the supporting matrix to yield a more efficient exploration pattern. Latrophilins have been reported to interact with intracellular scaffolding proteins known to be associated with the actin cytoskeleton but the functionality of such interaction remained elusive (Figures 2A–C). Recent data from our lab monitoring the formation of actin-rich structures evidenced an active role for latrophilins in regulating the formation of filopodia and lamellipodia (Figures 2A,B; Cruz-Ortega and Boucard, 2019). While all isoforms of latrophilins led to a constitutive activation of cofilin, which is an important modulator of actin rearrangement, isoform-specific functions were detected in the genesis of cell protrusion in response to teneurin binding (Cruz-Ortega and Boucard, 2019). Importantly, teneurin signals removed Lphn-induced inhibition on cell protrusions formation leading to an increase in filopodia. Interestingly, teneurin C-terminal peptides have been shown to activate small GTPases that contribute directly to the formation of actin structures and to act through latrophilins to modify actin dynamics (Chand et al., 2012; Husic et al., 2019). Thus, we hypothesize that latrophilins provide a framework for the establishment of adhesion structures by interacting with the actin cytoskeleton machinery (Figures 2A,B), a role that might precede the formation of adhesion complexes at the synapse. Molecular adhesion events via latrophilin-teneurin interactions would therefore act as permissive signals allowing intercellular contacts to establish a given adhesive structure.

### Latrophilins and Ligands: Molecular Aspects Involved in Synapse Formation and Function

The immense diversity of neuronal connections begs for a molecular code that can sustain such a high level of heterogeneity. Because adhesive properties of neurons are mainly embodied by cell adhesion molecules (CAMs), the biological support for heterogeneity should reflect an array of adhesion profiles supported by distinct sets of CAMs. However, it becomes clear that the shear number of adhesion molecule genes cannot by itself explain the diversity seen in neuronal connections. Thus, it is conceivable that the spatio-temporal patterns of established neuronal circuitry would be sculpted by the three following factors: (a) the types and forms of adhesion molecules expressed, (b) the net synaptic content of adhesion molecules, and (c) the pairing pattern of these adhesion molecules across synapses, in a given time frame throughout development. Thus, the genetic framework of neurons would have to provide the required information to produce a diverse array of proteins which can then carry the system’s heterogeneity on their shoulders. We will discuss how neuronal networks benefit from latrophilin and teneurin isoforms heterogeneity to generate adhesion complexes that are both diverse and hierarchical in nature.

#### Domain Modularity

Latrophilin has adjacent Lectin-like and Olfactomedin-like extracellular adhesion motifs separated by a short linker sequence which can be found inserted or absent from the translated protein as a result of alternative splicing of the corresponding transcribed mRNA. Thus, both these motifs are physically independent from each other and consequently, each domain is available to function independently with respect to their adhesion function. Indeed, the Lectin-like domain has been identified as the main interaction motif with teneurins, as its presence is absolutely necessary for latrophilin-1 to establish intermolecular complexes with teneurins (Boucard et al., 2014). On the other hand, it is completely dispensable when it comes to latrophilin-1 interacting with Neurexins or all latrophilins interacting with FLRT proteins. Conversely, the Olfactomedin-like domain represents the main interaction site for Neurexin and FLRT but is not essential for teneurins’ contact with latrophilin-1 as it serves modulatory purposes in this case by increasing affinity for the ligand-receptor pair. Important insights on ligand-receptor interaction were obtained from the first crystallographic determinations of a Lphn-ligand complex structure (Jackson et al., 2015, 2016; Lu et al., 2015). The Lphn-FLRT crystallographic complex revealed that the Lphn Olfactomedin-like domain forms a “rosette-like” structure of which the open face is engulfed within the concave face of FLRT LRR (Leucine Rich Repeats) horseshoe domain. Importantly, this domain is sufficient and necessary to form the required interaction with FLRT thus indicating that it can act in a modular fashion by restricting ligand binding to this region alone, leaving the other adhesion domain free to establish additional contacts. This interaction model is further supported by a recent study evidencing that latrophilins can form simultaneous complexes with FLRT and teneurins to generate different synaptic functions (Sando et al., 2019).

#### Alternative Splicing

**FIGURE 3:**
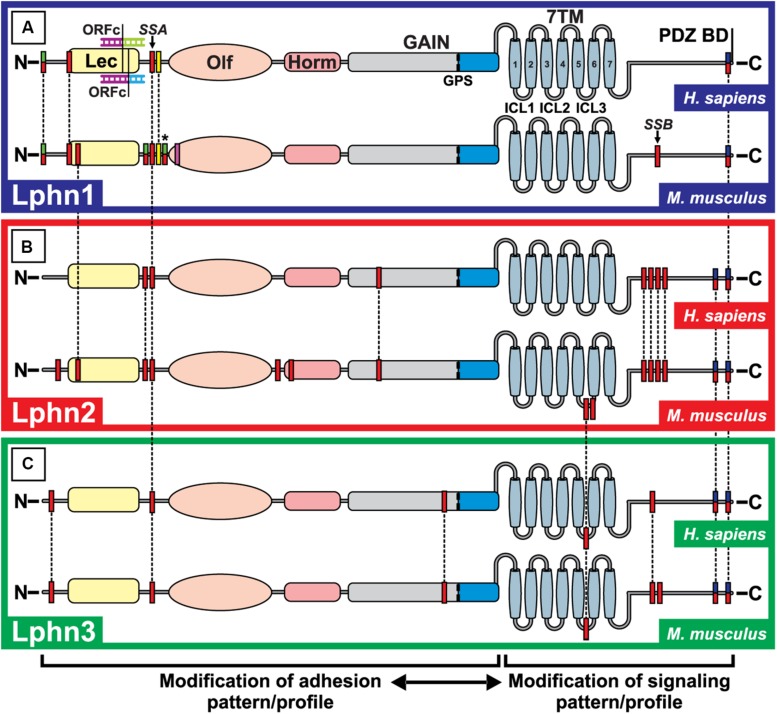
Alternative splicing events for Lphn1, 2 and 3 in *Homo sapiens* and *Mus musculus*. The potential alternative splicing events for Lphn1 **(A)**, Lphn2 **(B)** and Lphn 3 **(C)** are shown for *H. sapiens* and *M. musculus*. Each red box represents a possible splicing event. Green-red double colored boxes indicate splicing events which are only used to introduce a translation start site and thus cannot be combined within the same isoform, while the added asterisk indicates that these splicing events can alternatively be included as a continuous protein sequence of larger isoforms and can be combined with others of the same color code. The yellow box represents splicing events that are exclusively only present in the short isoforms lacking the Lectin domain. The pink box does not indicate a splicing event but represents the site of a potential alternative promoter sequence within a universally used exon. The Open Reading Frame Change (ORFc) indicates a splicing event present in the Lphn1 of H. Sapiens that alters the reading frame to generate a translation start site. Blue-red double colored boxes represent mutually exclusive splicing events (in the case of Lphn2 and Lphn3) two splicing sites with the same start sequence but which are carried out in different isoforms and are mutually excluding. The dotted lines indicate the common events between isoforms and species. The legend at the bottom indicates the possible consequences of the splicing events. Lec, Lectin domain; Olf, Olfactomedin domain; Horm, Hormone binding domain; GAIN, GPCR Autoproteolytic Inducing domain; GPS, GPCR proteolytic site; 7TM, Seven transmembrane domain; PDZ BD, PDZ binding domain; ICL1, ICL2, ICL3: Intracellular loop 1,2,3. ADGRL1,2,3, Adhesion G Protein-Coupled Receptor Latrophilin-1,2,3. Data were extracted from the NCBI database (www.ncbi.nlm.nih.gov) as well as the Ensembl database (www.ensembl.org). Primary assemblies for Homo sapiens and *Mus musculus*: GRCh38.p12 and GRCm38.p4. ADGRL1 Homo sapiens (Gene ID: 22859; NC_000019.10 and ENSRNOG00000072071); ADGRL1 *Mus musculus* (Gene ID: 330814; NC_000074.6 and ENSRNOG00000072071); ADGRL2 Homo sapiens (gene ID: 23266; NC_000001.11 and ENSG00000117114); ADGRL2 *Mus musculus* (Gene ID: 99633; NC_000069.6 and ENSMUSG00000028184); ADGRL3 Homo sapiens (Gene ID:23284; NC_000004.12 and ENSG00000150471); ADGRL3 *Mus musculus* (Gene ID: 319387; NC_000071.6 and ENSMUSG00000037605) (National Center for Biotechnology Information, 1988; Zerbino et al., 2018).

As a strategy to generate a high order of multiplicity in inter-neuronal contacts, adhesion molecules expressed in the nervous system display multiple variants originating from alternative splicing. The physiological importance of splicing events for neuronal functions such as synapse identity or maturation is best exemplified by Neurexins, Down Syndrome Cell Adhesion Molecule (DSCAM) and protocadherins which can produce from 3,000 to 40,000 variants, each with potentially different binding/adhesion functions (Wu et al., 2012; Treutlein et al., 2014; Kostadinov and Sanes, 2015). Latrophilin mRNAs are prone to multiple events of alternative splicing that show a certain level of heterogeneity between isoforms (Sugita et al., 1998; Matsushita et al., 1999). These splicing events create receptor variants that differ in their extracellular and/or their intracellular regions (Figure 3).

##### Extracellular splice inserts

The splicing pattern of latrophilins paints a complex portrait depicting isoforms with alternative initiation sites and intra-exonic splicing events (Figure 3). To this date, the impact on latrophilins of all extracellular splicing events are unknown except for one designated as splice site A (SSA). SSA is located in a region corresponding to the N-terminal extracellular portion of latrophilins, is common to all mammalian latrophilin isoforms and introduces or removes a 4–5 amino acid sequence between the Lectin and Olfactomedin domains (Sugita et al., 1998; Boucard et al., 2014). Lphn2 SSA variants display a slight variation from Lphn1 and Lphn3 SSA since they can include two variations of this insert, one that is identical to the other isoforms SSAs and another shorter form that differ in its N-terminal residues (Boucard et al., 2014). Splicing at SSA has been shown to modulate Lphn1-teneurin2/Lphn1-teneurin4 interactions such that the presence of this insert decreases their binding affinity (Boucard et al., 2014). Interestingly, this splicing-dependent modulation of affinity is specific to the Lphn1-teneurin pairs as Lphn1-Neurexin/FLRT pairings did not display a significant change to their binding properties whether the SSA insert was present or not. The function of the additional latrophilin-2 and -3 extracellular splicing events are unknown but it is likely that they may also contribute to ligand selection or receptor activation paradigms by stabilizing different conformations (Figure 3).

##### Intracellular splice inserts

The splicing events affecting the intracellular portions of latrophilins describes a rather complex pattern. In contrast to SSA splicing, a partial overlap has been observed between Lphn2 and Lphn3 variants while most Lphn1 splicing variants are unique to this isoform. The Lphn1 intracellular splicing site B, SSB, inserts or removes a 45 amino acid domain in the C-terminal tail of mouse Lphn1 but has not been detected in human Lphn1 (Figure 3A); Lphn2 and 3 are modified in their third intracellular loop and in their C-terminal tail at a site different from Lphn1 SSB and which displays a tandem splicing pattern (Figures 3A–C; Sugita et al., 1998). The function of these splicing events is still elusive; however, a recent study suggests that they could have a role in modulating intracellular signaling pathways such as functional coupling to G proteins (Rothe et al., 2019).

##### Regulation of alternative splicing

How these splicing events are regulated is not understood to this date. It is likely that various splicing factors are involved in this process given the complexity of the events observed. No splicing factors have been identified so far but evidences suggest that these events of alternative splicing are highly regulated. Indeed, while the relative expression of receptor variants resulting from mRNA splicing does not seem to vary according to different development stages in a given tissue, it differs between tissues at a given developmental stage. For example, the insert in SSA that is unique to Lphn2 is inserted in 60% of transcripts from the brain but is almost inexistent in transcripts from heart tissues of adult mice (Boucard et al., 2014). Moreover, the respective proportion of receptor variants in a given tissue varies between isoforms: the main Lphn3 isoform in brain does not contain SSA insert while Lphn1 SSA is present in approximately 50% of brain transcripts (Boucard et al., 2014). Thus, cell environments might be the dominating factor in determining which splicing variants of latrophilin will be generated.

##### Alternatively spliced latrophilin ligands

The molecular counterparts of latrophilins also exhibit alternative splicing that affects specificity of interaction with these adhesion GPCRs.

− Teneurins can be spliced in two extracellular sites: one within the EGF repeats region and another in the β-propeller region. The teneurin splice variant containing an insert in the β-propeller site loses its ability to form intercellular adhesion complexes through latrophilins, an effect that could be attributed to structural rearrangements rather than direct perturbation at the binding interface because this site is remote from where the binding occurs with latrophilins (Silva et al., 2011; Li et al., 2018). Interestingly, this variant of teneurin which is deficient in latrophilin binding loses the ability to induce post-synaptic excitatory specializations, instead it stabilizes inhibitory synapses, which suggests that latrophilins might not be the exclusive binding partners of teneurins. Indeed, Berns et al. (2018) described a strict homophilic interaction between teneurin-3 splice variants which specifically involve the variant which cannot bind to latrophilins (includes the splice insert in β-propeller site).

− Neurexins form a family of highly polymorphic proteins due to alternative splicing involving 5 sites for α-neurexin (SS1–SS5) and two for β-neurexins (SS4–SS5) (Treutlein et al., 2014). The binding of neurexins to their canonical ligand neuroligin is partly regulated by neurexin splicing at SS4 such that presence of an insert in this site decreases its affinity for neuroligin (Boucard et al., 2005). A similar pattern of interaction was observed for neurexin binding to latrophilin-1 although resulting in a complete abrogation when SS4 was present (Boucard et al., 2012). Importantly, both latrophilin-1 and neuroligin compete for the same binding pocket on SS4-deficient neurexin which makes them mutually exclusive in eventual molecular complex formation centered in Neurexins. This binding characteristic might explain why we and others were not successful in our attempts to directly isolate latrophilin-Neurexin complexes from brain extracts which contain high amounts of neuroligins (Boucard et al., 2005, 2014; Silva et al., 2011).

## *Cis*- Versus *Trans*-Interactions: Relevance for Ligand-Dependent Latrophilins’ Function

Inter-neuronal adhesion functions of latrophilins are primarily thought to occur via interactions in *trans*, i.e., latrophilins from one neuron interact with ligands expressed in another neuron. This interaction model infers that latrophilins would be restricted to one synaptic compartment in order to link another synaptic compartment displaying its ligands at the cell surface, thus fulfilling their role as *de facto* adhesion pairs. However, is it possible for latrophilins to participate in adhesion if they form complexes in *cis*, i.e., with adhesion molecules expressed in the same cells. We address three questions: is latrophilin pre- or post-synaptic? on which synaptic compartment are the latrophilin ligands present? which of these two types of interaction (*cis* versus *trans*) is functional?

The answer to the first question as to if latrophilins are pre- or post- synaptic relies on the following evidence: (1) The presynaptic neurotransmitter release machinery is activated when α-latrotoxin acts through latrophilin; (2) Electron microscopy with immunodetection of latrophilin’s extracellular domain detected an enrichment in the pre-synaptic membrane (Silva et al., 2011); (3) Growth cones, which can be conceptually seen as immature presynaptic structures, respond to latrophilin ligand teneurin to follow their course and acquire directionality (Vysokov et al., 2018); (4) A presynaptic phenotype was observed when knocking down Lphn isoforms (O’Sullivan et al., 2012). On the other hand, there is also evidence for post-synaptic localization of latrophilin: (1) Latrophilins can form a complex with proteins from the SHANK family predominantly expressed in the postsynaptic compartments of excitatory synapses (Kreienkamp et al., 2000; Tobaben et al., 2000); (2) Conditionally expressed Lphn2 and 3 fusion proteins colocalize with postsynaptic markers in the hippocampus (Sando et al., 2019); (3) Mouse models of Lphn2 and 3 deficiency display postsynaptic phenotypes in neurons of the hippocampus (Anderson et al., 2017; Sando et al., 2019). It is unclear whether Lphn are enriched in a given synaptic compartment, however, it appears conceivable that these receptors would be present in both, perhaps depending on the given developmental stages or neuronal types.

**FIGURE 4:**
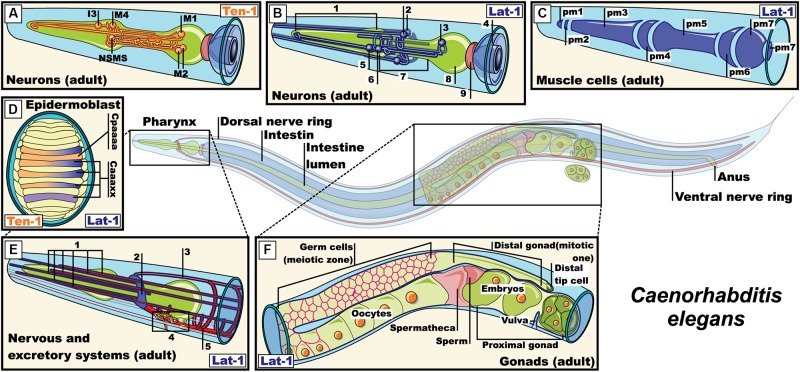
Expression pattern of lat-1 and teneurin-1 in *Caenorhabditis elegans.* **(A)** Expression of teneurin-1a in neurons M1, M2, M4, I3, NSMs of the pharyngeal nervous system (Morck et al., 2010); **(B)** Expression of lat-1 in pharyngeal and extra-pharyngeal neurons near of terminal bulb and isthmus that belong to the pharyngeal nervous system (**1**, *Corpus*; **2**, *Extra-pharyngeal neurons*; **3**, *Neuron in the terminal bulb*; **4**, *Intestine*; **5**, *Pharyngeal neurons*; **6**, *Neuron in the corpus with projections into the isthmus*; **7**, *Isthmus*; **8**, *Terminal bulb*; **9**, *Pharyngeal-intestinal valve*) (Willson et al., 2004; Langenhan et al., 2009); **(C)** Expression of lat-1 in muscle cell membrane of the pharynx (Promel et al., 2012); **(D)** Representation of epidermoblast during dorsal intercalation. ten-1 expressed in Cpaaaa and Caaa lineages, while lat-1 expression is restricted to Caaa lineages (Promel et al., 2012); **(E)** Expression of lat-1 in excretory cells and neurons of the nerve ring (**1**, *Sensory dentrites*; **2**, *Nerve ring*; **3**, *Dorsal nerve cord*; **4**, *Excretory cells*; **5**, *Ventral nerve cord*) (Willson et al., 2004; Langenhan et al., 2009; Promel et al., 2012); **(F)** representation of the expression of lat-1 in gonads of an adult hermaphrodite (Langenhan et al., 2009; Promel et al., 2012). Pm: Pharyngeal muscle, NSMs: neurosecretory motor sensory. Images were adapted with the permission of Wormatlas (www.wormatlas.org).

**FIGURE 5:**
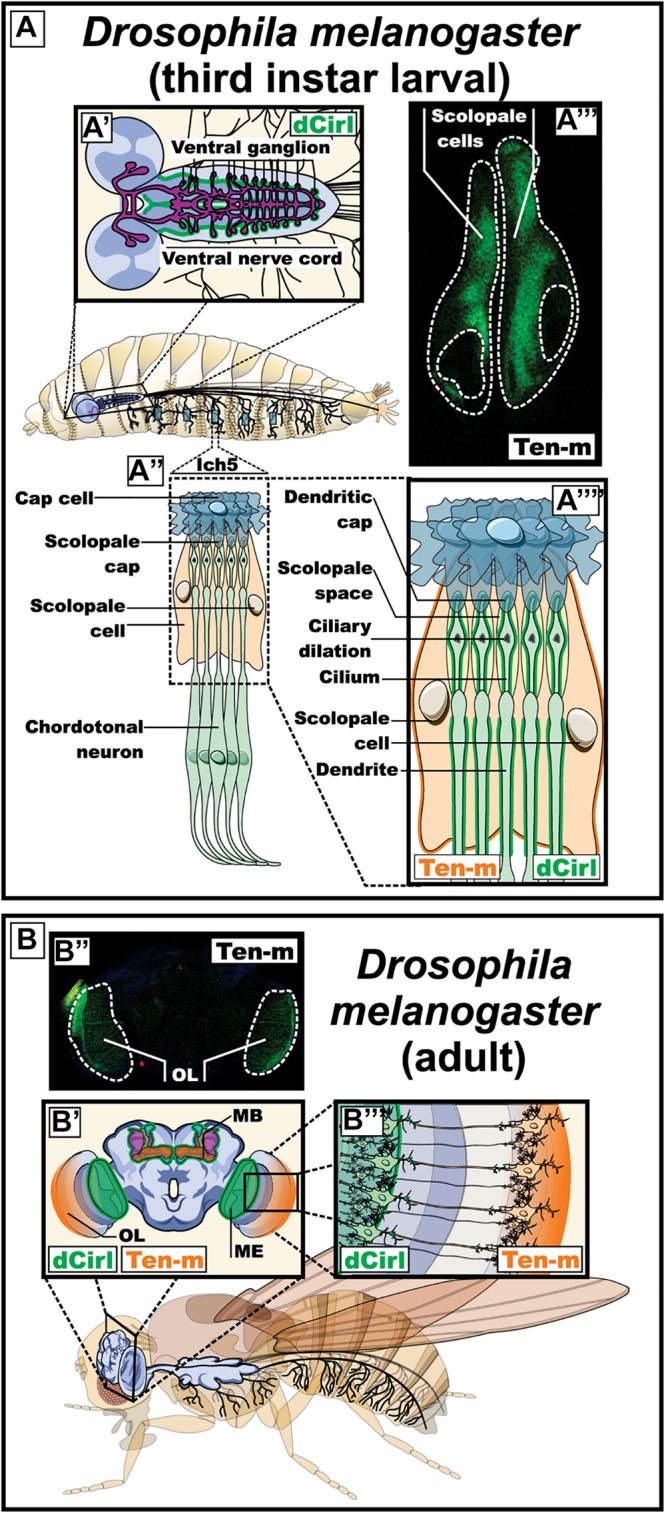
Expression pattern of *dCirl* and its possible ligand Ten-m in *Drosophila melanogaster* (fruit fly). **(A)** Expression of *dCirl* and Ten-m during the larval stage. A′) Schematic representation of the central nervous system indicating the reported expression of *dCirl* in the ganglion of the ventral nerve cord (in green) (Scholz et al., 2015). A″, A″″) Magnified representation of the sensory neurons in the pentascolopidial chordotonal organs (lch5) showing the reported expression pattern of *dCirl* in the dendritic membrane and the single cilium of chordotonal (ChO) neurons (in green) (Scholz et al., 2015). A″′) Fluorescence microscopy image showing Ten-m promoter-driven expression of YFP in the scolopale cells of the lch5 (in green). **(B)** Expression of *dCirl* and Ten-m during the adult stage. B′) Coronal view representation of the adult brain depicting *dCirl* expression in the medulla of the optic system and in the mushroom bodies (in green) (Gehring, 2014). B″) Fluorescence microscopy image showing Ten-m promoter-driven expression of YFP in the optic lobe (in green). B″′) Schematic representation of contacting photoreceptor neurons in the eye expressing Ten-m (in orange) and *dCirl* (in green) (Gehring, 2014). OL, optic lobe; MB, mushroom body; ME, medulla of the optic system.

The localization of latrophilin ligands (question 2) can be observed in both synaptic compartments. In mammals, teneurins are thought to participate in homophilic binding from both sides of the synapse, they are present in growth cones and shape neuronal circuits through axon guidance mechanisms (Nozumi et al., 2009; Young et al., 2013; Tran et al., 2015; Berns et al., 2018). In *C. elegans*, the neuromuscular expression of latrophilin (lat-1) and teneurin (ten-1) revealed a pattern of partly overlapping and complementary labeling suggesting both *cis* and *trans* configurations in the pharyngeal system with muscle/neuron lat-1/ten-1 complementarity along with neuron/neuron lat-1/ten-1 overlap (Figure 4). Non-neuronal systems from *C. elegans* suggest a *trans* configuration as exemplified by the epidermoblast stage. In this organism the establishment of an anterior (a) -posterior (p) axis is indispensable for cell polarity and future cell divisions. At the fourth division the Ca and Cp cells are generated according to their position in the anterior-posterior axis, respectively. In accordance with the above, the eigth division gives rises to Cpaaaa cells expressing ten-1 which are surrounded by Caaa lineage cells expressing lat-1, thus depicting a *trans* configuration. However, at this cell division stage, a *cis* configuration is also supported giving the concomitant expression of both proteins in Caaa lineages (Figure 4D; Langenhan et al., 2009; Promel et al., 2012). In *Drosophilia*, teneurin orthologs participate in axonal pathfinding thus evidencing their role in both sides of contacting membranes (Hong et al., 2012; Mosca et al., 2012). In an attempt to probe the *Drosophila* teneurin (Ten-m) expression pattern, we conducted experiments using a promoter enhancer trap that allowed for the visualization of enhanced yellow fluorescent protein (eYFP) driven from the Ten-m promoter, relying on the Gal4-UAS system (Figure 5; Hacker et al., 2003; Horn et al., 2003). We observed a complementary expression pattern with the reported *Drosophila* latrophilin (*dCirl*) expression at the larval stage in the chordotonal organ (Figure 5). Indeed, while *dCirl* has been reported to be expressed in chordotonal neurons (Scholz et al., 2015), our analysis revealed that Ten-m was expressed in the adjacent scolopale cells, thus suggesting a *trans* configuration (Figure 5). Moreover, the expression of *Ten-m* in the optic lobe of the adult *Drosophila* brain was detected in photoreceptor neurons that project to the medulla where *dCirl*-expressing neurons have been identified (Figure 5; Gehring, 2014). Although the Ten-m/*dCirl* interaction in *trans* has yet to be reported in *Drosophila*, our data point to a complementary pattern of expression that is suggestive of a *trans* configuration in this organism’s sensory organs.

As the possibility of many configurations of interactions seems more than likely, we are left with the third issue, which is bound to capture further attention: can the functionality of *cis* interactions rival the one from *trans* interactions? Since latrophilins and their ligands are transmembrane proteins (except for contactin-6), each can elicit an intracellular signal independently of their respective partnership. On one hand, the hypothesis of *trans* configuration calls for a system that segregates anterograde and retrograde signaling in different cells, thus generating a “one-ligand-one-signal” environment in regards to individual contacting cells. On the other hand, the *cis* configuration has the potential to create intersecting signaling pathways. Evaluating the functionality of both configurations, Li et al. (2018) observed that teneurin-2 was capable of inducing a similar decrease in cAMP accumulation in cells expressing Lphn1 and Lphn3 whether expressed in the same cell or on different cells. Because latrophilins are known to couple to G proteins (Li et al., 2018; Rothe et al., 2019) from which the β/γ subunits, once dissociated from the α subunit, can in turn activate the MAPK pathway, it remains to be seen if the teneurin-dependent activation of the FAK pathway would affect MAPK signaling differently in a *cis* versus a *trans* configuration (Suzuki et al., 2012, 2014). The functional impact of these configurations will have to be assessed in future studies in order to grasp the full understanding of their physiological or pathophysiological implications and to test whether the functional considerations we propose as a hypothesis, are valid.

## Latrophilins and Systemic Functions in Model Organisms

The physiological functions of latrophilins have been investigated in multiple organisms. The accumulating data point to an evolutionary conserved role while simultaneously demonstrating divergences. We will detail observations emanating from the study of latrophilin deficient animals in order to highlight overlapping as well as non-overlapping latrophilin functions.

### Latrophilins in the Nematode Worms

The two known orthologs of latrophilins in *Caenorabditis elegans* were named lat-1 and lat-2 (Mee et al., 2004; Willson et al., 2004). These proteins lack an olfactomedin domain at their amino terminal end, which differentiates them from their mammalian orthologs (Mee et al., 2004; Willson et al., 2004). During the early stages of the nematode’s life cycle, lat-1 is expressed in the gonads during oogenesis, in blastomeres (especially in those derived from the AB lineage) and pharyngeal and epidermal precursors (Figure 4; Langenhan et al., 2009). During the larval and adult stage, its expression has been reported in the vulva, plasma membrane of pharyngeal cells, in neurons of the terminal bulb and the corpus (with projections inside the isthmus) and in neurons of the nervous ring (Willson et al., 2004; Langenhan et al., 2009; Promel et al., 2012). On the other hand, the expression of lat-2 overlaps with lat-1 in the pharyngeal primordium during the early stages and is limited to cells of the excretory and pharyngeal system in the larval and adult stages (Figure 4; Langenhan et al., 2009). lat-1, but not lat-2, is required for proper development during the early stages of embryogenesis, specifically for regulating the alignment of the anterior and posterior planes during the fourth round of cell division through its coupling with Gα_S_ proteins. This coupling fostered the activation of adenylate cyclase, increasing the intracellular levels of cAMP in wild-type embryos, which were decreased in lat-1 knockout embryos (Langenhan et al., 2009; Promel et al., 2012; Muller et al., 2015).

The *C. elegans* pharynx is a neuromuscular feeding organ that is related to the transport of food from the mouth to the intestine through pharyngeal pumps and isthmus peristalsis (Albertson and Thomson, 1976; Trojanowski et al., 2016). These relaxation-contraction cycles are regulated in part by neurotransmitters such as acetylcholine, serotonin and glutamate from neurons of the pharyngeal and extra pharyngeal nervous system, and by myogenic activity (Bhatla et al., 2015; Trojanowski et al., 2016). The main motoneurons that regulate pumping are the cholinergic neurons MCs and glutamatergic M3 neurons, which connect with pm4 muscle cells of the metacorpus (Figure 4). When food is present in the environment, neurosecretory motoneurons (NSM) start secreting serotonin to activate MCs and M3 which in turn release acetylcholine and glutamate, respectively, on cells of the pharyngeal muscle, thus regulating the duration of the food intake circuit. Ablation of MCs and M3 neurons led to a decrease in the number of pharyngeal contractions and interestingly, so did the lat-1 knockout and knockdown models (Avery and Horvitz, 1989; Willson et al., 2004). In order to investigate how lat-1 expression in pharyngeal cells and nearby neurons relate to the neural network that regulates pumping during food intake, lat-1 knockout worms were treated with the serotonin reuptake inhibitor imipramine or the anthelmintic emodepside acting at the neuromuscular junction, observing a resistance to their effect compared to wild-type worms (Mee et al., 2004; Willson et al., 2004). Thus, serotonin and acetylcholine may mediate lat-1 function in worms. However, another question arises: could lat-1 adhesion function from the pharyngeal muscle be completed by another adhesion molecule located in pharyngeal neurons? Teneurins come to mind as potential candidates giving their similarities to their mammalian orthologs. In *C. elegans*, a single gene has been reported that transcribes two isoforms of teneurin: teneurin 1-L (large) and 1-S (small, because it lacks the intracellular domain). While both isoforms share overlapping expression profiles in the nervous system from embryonic stages to the adult stage, teneurin 1-L is also expressed in intestinal cells and the pharynx (Figure 4). Because neurons that express teneurin 1-L are part of the circuit that regulates the pharyngeal pumping (M1–M4, I3, and NSM), it is tempting to speculate that a lat-1/ten1 complex could be mediating neuromuscular functions related to pharyngeal pumping.

### Latrophilin in Flies

The dipteran fly *Drosophila melanogaster* is an accessible model organism for scientific research, arguably the multicellular organism understood in most detail. Genetic, cellular and molecular tools have been developed in over a century of continuous genetic and biological research in this model (Yamaguchi and Yoshida, 2018). Its genome is comparatively small, however, many genes, as well as principles and mechanisms of development, are evolutionarily conserved in vertebrates (Adams et al., 2000). *Drosophila* neurons and glia are no exception and share many molecular and functional characteristics with the related cell types in mammals (Venkatasubramanian and Mann, 2019; Yildirim et al., 2019). Neuronal axons have all the machinery necessary to transmit nerve impulses in a similar way to how action potentials are generated in mammals leading to neurotransmitter release at the synapses (Jekely et al., 2018; Kasture et al., 2018; Rich and Terman, 2018; Sugie et al., 2018). Hence, *Drosophila* offers a good model to study neuronal proteins (Monnier et al., 2018; Rosas-Arellano et al., 2018). *Drosophila melanogaster* only has a single homolog of latrophilins (Scholz et al., 2015). The single homolog of latrophilins in this species is known as *dCirl*, expressed during the larval stage in peripheral sensory neurons, including those of the pentascolopidial chordotonal organs (lch5), and in the ventral nerve cord (Figure 5; Scholz et al., 2015). *dCirl* shows a strong expression pattern in the dendritic membrane and the single cilium of chordotonal (ChO) neurons of lch5. The mature lch5s are composed of multicellular units called scolopidia; each unit consists of three bipolar neurons and support cells. The distal segment of each dendrite in these neurons ends in a cilium that is protected by the supporting scolopale cell. The lch5 is in charge of mediating the mechanosensation process by means of which the mechanical stimuli such as touch, hearing and mechanical deformation of the larval body during the locomotion induce the movement of the ciliated dendrites causing the opening of cationic channels and an inrush of K^+^ leading to a neuronal depolarization that is translated into neuronal impulses (Prahlad et al., 2017).

A null *dCirl* mutation demonstrated that this gene is not essential for development and viability. The mutant organisms did, however, manifest obvious alterations in their sensory organs: the structures most affected were the lch5s (Scholz et al., 2015). The *dCirl* knockout larvae showed abnormal behaviors, such as a conspicuous crawling pattern and traveling less distance than control larvae. These results suggested that *dCirl* participates in shaping locomotion. In addition, the *dCirl* mutants showed diminished touch sensitivity, as well as a reduction in mechanosensory responses after mechanical stimuli. All these alterations were corrected after the re-expression of *dCirl* in the ChO neurons, which showed that the observed effects were due specifically to the loss of *dCirl*. On the other hand, the morphology of ChO neurons was not altered after the removal of *dCirl*, suggesting that *dCirl* plays a functional rather than a structural role in these neurons, being required for adequate sensitivity gentle touch, sound and proprioceptive feedback during larval locomotion (Scholz et al., 2015). The mechanism by which *dCirl* is thought to relay mechanotransduction was investigated through genetic interaction assays which revealed that two subunits from Transient Receptor Potential (TRP) channel, TRPN1/NompC and TRPV/Nanchung (Kim et al., 2003; Cheng et al., 2010), could mediate its function possibly by providing the ion flux necessary for the decoding of the mechanical strain by generating a receptor potential in mechanosensory neurons (Scholz et al., 2015).

An interesting observation was made when intracellular signaling of *dCirl* was investigated using a FRET- based cAMP sensor. Mechanostimulation of *dCirl* decreased the concentration of cAMP in mechanosensory neurons (Scholz et al., 2017). *dCirl*-deficient flies did not display a reduction in cAMP upon mechanostimulation and consequently experienced a quenching of neuronal activity. These observations suggest that *dCirl* modulates neuronal activity by suppressing cAMP production, a signaling feature that reveals a stark contrast with lat-1 signaling in C. elegans (see section “Latrophilin in the Nematode Worms”). This difference could be accounted for if *dCirl* and lat-1 are coupled to distinct G_α_ subunits. Conversely, the deficiency observed in *dCirl*-deficient flies could be rescued by pharmacological inhibition of adenylate cyclase (Scholz et al., 2017). The role of *dCirl* in the lch5 of *Drosophila* as a mediator of mechanosensation represents a novel function for this family of receptors and highlights the importance of conformational changes for its ability to trigger intracellular signaling cascades, a feature resembling canonical activation mechanisms of members of the GPCR family (Oldham and Hamm, 2008).

In the adult brain *dCirl* expression was observed in the medulla of the optic system and in the mushroom bodies, the latter of great importance for olfactory learning and memory in *Drosophila* (Figure 5; Gehring, 2014; Guven-Ozkan and Davis, 2014; Hige, 2018). The latrophilins have been associated with various neuropsychiatric diseases, among those Attention-Deficit Hyperactivity Disorder (ADHD) has received particular attention, since multiple studies associate the Lphn3 gene with the etiology of the disease (discussed below) (Arcos-Burgos et al., 2010; Domene et al., 2011; Ribases et al., 2011; Jain et al., 2012; Labbe et al., 2012; Fallgatter et al., 2013; Acosta et al., 2016; Kappel et al., 2017; Huang et al., 2018). A conditional *dCirl* knockdown model based on RNA interference was generated in *Drosophila* (van der Voet et al., 2016). Neuronal-specific decrease in *dCirl* expression induced hyperactivity and reduced average sleep time during the night (dark) phase providing evidence that the Dopamine-related paradigm for latrophilin function is also conserved in *Drosophila*.

As mentioned, a potential endogenous ligand for *Drosophila* latrophilin is the teneurin Ten-m, a transmembrane protein with a documented role in synapse formation in this organism (Mosca and Luo, 2014). Using a reporter line, we assessed the expression of *Ten-m* in the *Drosophila* larva (Figure 5). Expression was observed in the bolwig organ (the larval eye) neuron, projecting into the optic lobes of the brain and in the lch5 organs, but, in contrast to *dCirl* expression in the neurons, *Ten-m* is observed in the surrounding scolopate cells (Figure 5 and Supplementary Figure S1). In the adult fly, we detected Ten-m promoter expression in the brain optic lobes in a pattern that stopped at the medulla right where the expression of *dCirl* was reported (Figure 5). These results suggest the possibility of a *trans*-synaptic contact between *dCirl* and Ten-m in *Drosophila*, which remains to be tested experimentally.

### Latrophilin in Zebrafish

*Danio rerio*, commonly known as zebrafish, is one of the model organisms for vertebrates used in scientific research, particularly in studies that address different aspects of neurogenesis. Among the advantages offered by this organism are that they have an external development accessible to experimental manipulation, as well as a rapid development of the larval nervous system, which is established within 4 days of development. Additionally, the zebrafish has also been used for behavioral studies, since it is a diurnal and naturally sociable animal that shows preferences for community life. Currently a large amount of genetic and anatomical information of zebrafish is available in databases, which facilitates studies using this organism as a model (Kuwada, 1995; Norton and Bally-Cuif, 2010; Schmidt et al., 2013).

**FIGURE 6:**
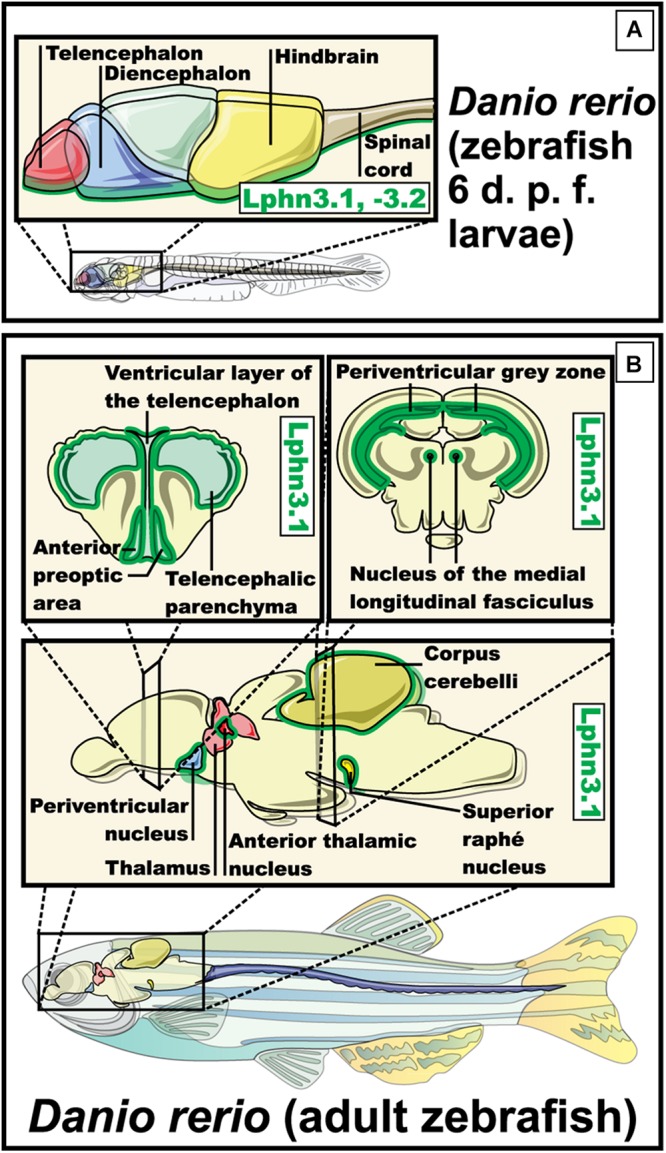
Expression pattern of Lphn3.1 – Lphn3.2 in *Danio rerio* (zebrafish). **(A)** Expression of the two orthologs for lphn3 in zebra fish, both present a similar expression pattern during the larval stage observed in the ventral part of the telencephalon and diencephalon, hindbrain, the posterior brain and the ventral area of the spine (in green) (Lange et al., 2012). **(B)** Expression in adult zebrafish of Lphn3.1 is shown along the telencephalic midline, telencephalic parenchyma, the anterior thalamus, the periacal ductal gray matter, the superior nucleus of raphe, the periventricular nucleus of the lower hypothalamus, the cerebellum and the nucleus of the medial longitudinal fascicle (green outline) (Lange et al., 2012).

Due to its characteristics the zebrafish was used to study the development and function of latrophilins. The zebrafish has two orthologs for the isoform 3 of the latrophilins, which are called Lphn3.1 and Lphn3.2. Both orthologs present a similar expression profile during development. As the larval maturation progresses, Lphn3.1 and Lphn3.2 display a shared expression pattern becoming more prominent in the ventral part of the telencephalon and diencephalon, in the posterior brain and in the ventral area of the spine (Figure 6). In the brain of adult zebrafish the expression of Lphn3.1 is detected along the telencephalic midline, as well as in lower levels in the telencephalic parenchyma, the anterior thalamus, the periacal ductal gray matter, the superior nucleus of raphe, the periventricular nucleus of the inferior hypothalamus, the cerebellum and the nucleus of the medial longitudinal fascicle. Because Lphn3.1 expression profile coincided with the expression of its murine ortholog, this receptor′s function in zebrafish was further investigated (Lange et al., 2012). Lphn3.1 knockdown morphants increased their swimming distances and displayed hyperactivity, a phenotype that has been associated with the dysfunction of Lphn3 gene in humans affected by ADHD. However, careful considerations should be taken when comparing different organisms because of existing differences in neuroanatomy and circuit formation (Lange et al., 2012; Akutagava-Martins et al., 2016). Such variations are well exemplified in the dopaminergic system of zebrafish. In this model organism, the major dopamine (DA) regions are olfactory bulb, preoptic region, pretectum, posterior tuberculum and hypothalamus. This pattern differs with mammals mainly because no DA neurons are found in the mesencephalon of the zebrafish (Schweitzer and Driever, 2009). Among other things, DA helps to regulate movement, which was altered in Lphn3.1 morphants, leading to the hypothesis that reduction of Lphn3.1 expression influences the dopaminergic system in some way. However, when the concentrations of DA and its metabolite 3,4-dihydroxyphenylacetic acid were evaluated in whole larvae, no significant difference was found compared to the controls (Lange et al., 2012).

One of the structures involved in the control of locomotion in zebrafish is the posterior tuberculum, a structure that corresponds to one of the regions with the highest number of DA neurons in the brain of zebrafish. Interestingly, disorganization, as well as a reduction in the overall number of neurons in the posterior tuberculum was observed in Lphn3.1 morphants. These organisms also showed a reduction in the total number of DA neurons in this structure (Schweitzer and Driever, 2009; Tay et al., 2011; Lange et al., 2012).

The characterization of Lphn3.2 remains elusive and it is tempting to speculate on their level of redundancy in zebrafish physiology. However, the studies conducted in zebrafish show the importance of Lphn3.1 in the control of movement and provide a clue of its relationship with the dopaminergic system.

Teneurin homologs reported in zebrafish brain include Ten-m3 and Ten-m4, as potential Lphn3 ligands. In the forebrain and the midbrain, Ten-m3 and Ten-m4 have a complementary expression: Ten-m3 is expressed in the optic vesicles, the region covering the caudal diencephalon and the mesencephalon showing strongest expression at its most anterior part, while Ten-m4 is expressed in the rostral diencephalon with the least expression in the optic vesicles, and a region covering the mesencephalon and the midbrain/hindbrain boundary (Mieda et al., 1999). Unlike their mammalian counterparts, there are no reports of an interaction between orthologs of latrophilins and homologs of teneurins in zebrafish (Boucard et al., 2014).

### Latrophilins in Mice

Mice genomes express three isoforms of latrophilins: Lphn1, Lphn2 and Lphn3. While enriched in neurons these receptors can also be found expressed in non-neuronal tissues such as kidney, lung and heart.

#### Synaptic Phenotypes

**FIGURE 7:**
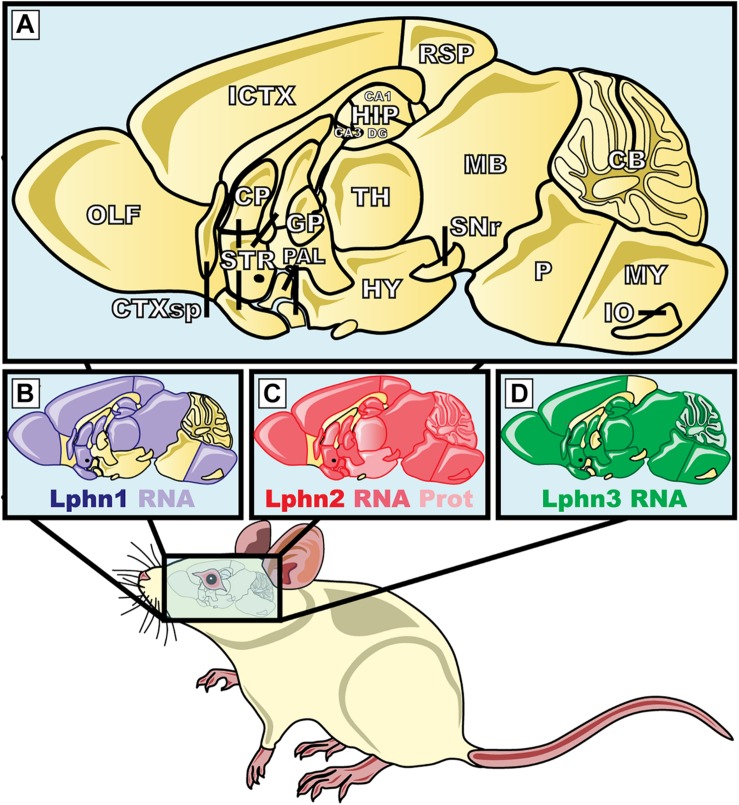
Expression pattern of the three isoforms of latrophilins in the central nervous system of *Mus musculus*. **(A)** The magnification represents the mid-sagittal section of the brain of *M. musculus*; **(B–D)** areas highlighted in purple, red and green show the latrophilin-1, 2, and 3 mRNA or protein expression, respectively. ICTX, Isocortex; HIP, hippocampus; CA, Ammon′s horn; DG, dentate gyrus; HY, hyppothalamus; CP, caudate puntamen; TH, thalamus; IO, inferior olivary; STR, striatum; SNr, substantia nigra pars reticulata; GP, globus pallidus; RSP, retrosplenial cortex; CB, cerebellum, CTXsp, cortical subplate; MB, midbrain; MY, medula; OLF, olfatory areas; PAL, pallidum; P, pons. Protein data were from Anderson et al. (2017). mRNA data were extracted from Kreienkamp et al. (2000), Tobaben et al. (2000) and the Allen Brain Atlas (www.brain-map.org).

*Lphn1-* Latrophilin-1 is the most abundant isoform expressed in the adult mouse brain (Figure 7; Sugita et al., 1998; Matsushita et al., 1999; Boucard et al., 2014). Despite this early observation very few studies report on the role of this isoform in central synapses. An indirect assessment of its synaptic role obtained through the use of α-Latrotoxin on isolated synaptosomes of mice lacking Lphn1, revealed that this receptor isoform participated in glutamate release (Tobaben et al., 2002).

*Lphn2-* This isoform appears to be widely expressed, thus showing a widespread presence in many neuronal cell types (Figure 7; Kreienkamp et al., 2000; Anderson et al., 2017). Despite latrophilin-2 being potentially present in many types of synapses, its predominant function was observed to contribute to the development of specific synaptic sites. A study conducted by Anderson et al. (2017) aiming to characterize the role of latrophilin-2 in the synaptic physiology of the hippocampus found that this presumptive receptor for α-latrotoxin played a post-synaptic role rather than a pre-synaptic one, at least in the system surveyed. In the hippocampus neuronal network, neurons from the entorhinal cortex send projections to the CA1-region pyramidal neurons of the stratum lacunosum-moleculare (SLM), thus representing the pre- versus post- synaptic configurations, respectively. Latrophilin-2 expression was found to be enriched in the SLM dendritic spines where its deletion led to post-synaptic defects of excitatory synapses linked to spine development and function whereas the properties and characteristics of inhibitory synapses where kept unchanged. Moreover, because the number of excitatory synapses was selectively reduced following a genetic deletion of Lphn2 in hippocampal neurons, the authors attributed this deficiency to the consequent alteration of the target recognition abilities of neurons lacking this receptor. Giving that the other Lphn isoforms are also expressed in the same neuronal network of the hippocampus, these data would suggest that the function of Lphn2 is not redundant in this network. This study provided unsuspected data pertaining to Lphn2 localization and function at synapses that raised the following questions amongst others: how is Lphn2 trafficked to both pre- and post- synaptic compartments? Which is the presynaptic ligand responsible for Lphn2 role in target recognition? To which extent can the function of Lphn2 be dissociated from the function of other Lphn isoforms? While the role of Lphn2 in neuronal physiology and function remain intriguing, one observation remains clear, Lphn2 is an essential element amongst the molecular determinants that support synaptogenesis in mammals.

*Lphn3-* As referenced in Section “The Role of Latrophilins in Human Neuropathophysiology,” this latrophilin isoform differs from the other isoforms because it amounts for most of the genetic associations made with human neurological disorders so far. While an assumption can be made for the role of Lphn3 in neuronal functions, its role at the synapse is far from being elucidated. Indeed, loss-of-function studies resulting in genetic deletion of Lphn3 in mice (*Mus musculus*) revealed that dopaminergic neurons as well as molecular determinants of the dopamine pathway were altered in genetically modified animals (Lange et al., 2012; Wallis et al., 2012; Orsini et al., 2016). The first piece of evidence indicating that Lphn3 might play a role at the synapse was provided by an RNA interference approach in which a reduction in Lphn3 mRNA levels in mice hippocampal neurons led to a defect in presynaptic function of excitatory neurons, an effect consistent to the receptor’s presumptive localization (O’Sullivan et al., 2012). The same RNA interference approach applied to cortical neurons *in vivo* provided a second line of evidence linking Lphn3 mRNA levels with the formation and function of specific synaptic contacts of the cortical circuitry (O’Sullivan et al., 2014). In this paradigm, the functional excitatory connection between cortical neurons from Layer 2/3 to Layer 5 neurons exhibited a postsynaptic defect with no measurable presynaptic defect while morphological determinants of this connection revealed both pre- and post-synaptic defects (O’Sullivan et al., 2014). The third line of evidence came from the study of Lphn3 conditional knockout mice which located Lphn3 expression to excitatory synapses of pyramidal CA1 neurons particularly enriched in stratum oriens (SO) and stratum radiatum (SR). These mice displayed defects in dendritic spines formation within SO and SR as well as a selective loss of excitatory synaptic inputs from Schaffer collateral projections, most recently many of these findings were replicated in a rat model (Regan et al., 2019; Sando et al., 2019). Thus, elucidating the function of Lphn3 will prove instrumental to understanding its role in physiological and pathophysiological brain functions.

#### Behavioral Phenotypes

*Lphn1*—Out of all latrophilin isoforms in mice, latrophilin-1 displays the higher expression in the central nervous system (Boucard et al., 2014). Despite this widely reported observation, no striking phenotype has been described for mice expressing a loss-of-function genotype for latrophilin-1. Other than a perceived lack of maternal instinct, Lphn1 deficient mice do not display severe behavioral defects and these mice are seemingly both viable and fertile in a manner that is indistinguishable from their wild-type counterparts (Tobaben et al., 2002).

*Lphn2*— This widely expressed latrophilin isoform appears to be essential for the proper development of mice. Indeed, constitutive Lphn2 deletion is embryonically lethal as litters from heterozygous crossing do not yield homozygous pups, thus hinting at a role that is most likely not neuron-specific but rather would refer to its importance in crucial developmental check points (Anderson et al., 2017). On the other hand, when Lphn2 is deleted in neurons only, mice suffer behavioral impairments that are linked to learning paradigms. Mice that lack Lphn2 in neurons possess less flexibility in the way they apply their learning abilities as they are unable to adapt to new learning paradigms that require a temporal change in a sequence of events (Anderson et al., 2017). These findings are particularly interesting in the context that these mice can learn tasks at a rate similar to their wild type counterparts because it suggests that Lphn2 would be required to allow generalized learning which supports the notions of abstraction or generalization, concepts that describe how a learning experience acquired in a particular context can then be applied when the context later changes by retaining core elements of learning.

*Lphn3*— The brain-enriched expression of this latrophilin isoform emphasizes its potential role in cognitive functions. Genetic manipulations leading to deletion of Lphn3 in the full organism causes marked alterations in behavior of engineered mice. A stark impediment in reward-seeking behavior can be observed in Lphn3-deficient mice as exemplified by a higher food consumption and a higher locomotor response to cocaine administration than their wild-type littermates (Wallis et al., 2012; Orsini et al., 2016). Additionally, these mice expressed a hyperactive phenotype measured in both horizontal and vertical activity with a concomitant higher level of stereotypy (Wallis et al., 2012). These behavioral phenotypes are reminiscent of traits elicited in addiction paradigms, thus suggesting that Lphn3 is required for regulating reward pathways.

## The Role of Latrophilins in Human Neuropathophysiology

**FIGURE 8:**
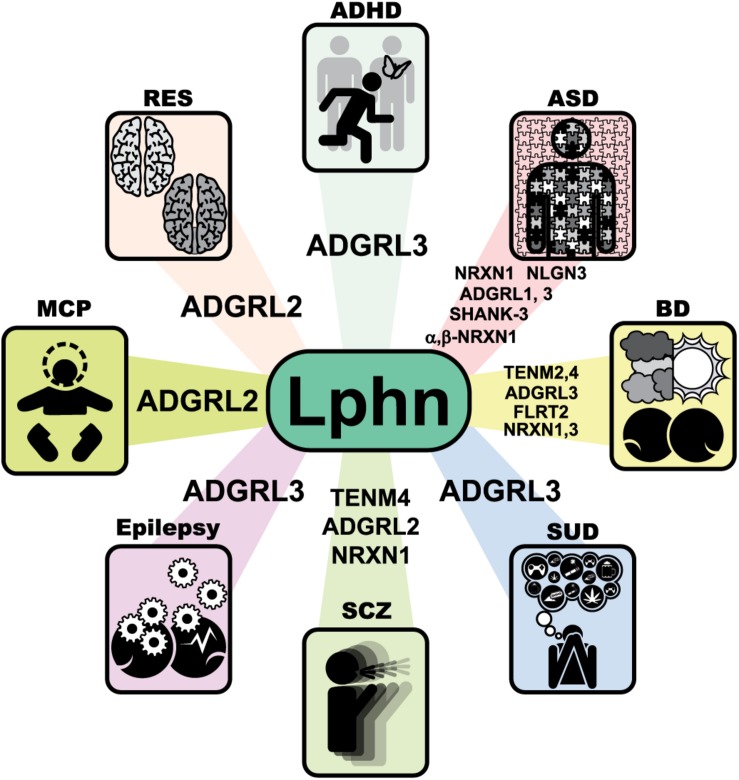
Molecular networks involving latrophilins and their ligands in human neuropathology. Neurological disorders associated with variations in genes from latrophilins and some of their endogenous ligands. Unless otherwise stated in the text, data were extracted from the *GWAS catalog* database and *Harmonizome* database (https://www.ebi.ac.uk/gwas/) (Buniello et al., 2019) and (http://amp.pharm.mssm.edu/Harmonizome/). ADHD, Attention Deficit Hyperactivity Disorder; ASD, Autism spectrum disorder; BD, Bipolar disorder; SUD, Substance use disorder; SCZ, Schizophrenia; MCP, microcephaly; RES, rhombencephalosynapsis.

According to the preferred expression of latrophilins in the brain, this family of receptors seems to be having an important role in this central decision making and executive organ. Thus, it is conceivable that modifications to the function of these GPCRs and/or their ligands will have repercussion in human health. Here we summarize a few neuronal disorders with which latrophilin genes defects have been associated such as: ADHD, substance use disorder (SUD), autism spectrum disorder (ASD), bipolar disorder (BD), schizophrenia (SCZ), epilepsy and microcephaly (MCP) (Figure 8).

### Attention Deficit and Hyperactivity Disorder (ADHD)

Attention deficit and hyperactivity disorder is a neurodevelopmental disorder that affects the brains cognitive functions and is characterized by a deficit of attention, hyperactivity and impulsivity. Its high level of heritability of approximately 75% suggests the involvement of a strong genetic components although some environmental factors are also suspected to influence the etiology of ADHD (Akutagava-Martins et al., 2016; Bonvicini et al., 2016). Due to the nature of the disease and its symptoms, the first genes to be studied associated with ADHD were part of the dopaminergic and serotonergic pathways, given that the neurotransmitters dopamine and serotonin are involved in attention, learning and motor control. Additionally, patients with ADHD are treated with medication that affect the transport of dopamine to the synapse or its retention or recapture by synaptic components but the mechanism by which these drugs act is not entirely clear (Faraone et al., 2005; Mick and Faraone, 2008; Genro et al., 2010).

In an effort to identify the genetic risk factors that contribute to the etiology of ADHD, a multigenerational study was carried out in an isolated population from Colombia with a high prevalence of ADHD. In this study, a significant link between ADHD and a region of chromosome 4q13.2 was reported and later circumscribed to the latrophilin-3 gene (ADGRL3) (Arcos-Burgos et al., 2010). Moreover, the presence of ADGRL3 single nucleotide polymorphisms (SNPs) were confirmed in other populations samples. A thorough analysis of ADGRL3 variants by gene sequencing led to the identification of polymorphisms in both exonic and intronic regions (Domene et al., 2011). Very few studies have addressed the ADHD-related ADGRL3 variations at the molecular level. One such study combining model organism genetics and *in vitro* assays identified an evolutionary conserved region located in a potential regulatory sequence within the minimal critical region attributed to ADGRL3. This region contained a three-variant ADHD risk haplotype (rs17226398, rs56038622, and rs2271338) that reduced the enhancer activity by 40%. One risk allele (rs2271338) was associated with a reduced expression of Lphn3 in the thalamus and the same risk allele was found to disrupt binding to the YY1 transcription factor, an important regulator of development of the central nervous system (Martinez et al., 2016).

Reinforcing the role of ADGRL3 in the etiology of ADHD, variants or haplotypes of this gene have been linked to the effectiveness of stimulant medication. However, the results obtained were controversial. On the one hand Arcos-Burgos et al. (2010) observed that the G allele carriers within ADHD-associated SNP rs6551665, presented a better response to medication regarding inattention whereas Labbe et al. (2012) reported that carriers of the same pathogenic allele displayed a lower response to treatment with respect to hyperactivity (Arcos-Burgos et al., 2010; Labbe et al., 2012). On the other hand, another study suggested that the homozygous carriers of the CGC haplotype (rs6813183, rs1355368, and rs734644) expressed a faster response to symptoms’ improvements following methylphenidate (MPH) treatment, a psychostimulant medication prescribed to alleviate symptoms of ADHD and thought to block dopamine reuptake (Volkow et al., 2002; Genro et al., 2010; Bruxel et al., 2015). However, a meta-analysis study of existing literature revealed that variant rs6551665 was not significantly associated with MPH response in children (Myer et al., 2018). The apparent discrepancies as to if ADGRL3 haplotypes represent clinically relevant predictors of treatment response could be attributed to differences in the ethnicity of the populations studied.

Little is known about the effects that environmental factors exert on the development of ADHD. Among known environmental factors, maternal smoking and stress during pregnancy are thought to increase the risk for developing ADHD. A significant association was detected between previously described ADGRL3 SNPs (rs6551665, rs1947274, rs6858066, and rs2345039) and MPH treatment after ADHD diagnosis under these environmental factors such that the patients which mothers experienced less stress had a better response outcome (Choudhry et al., 2012). However, another environmental factor associated with the use of acetaminophen during pregnancy did not yield a significant increase in ADHD-related symptoms in a model organism (Brandlistuen et al., 2013; Liew et al., 2014; Thompson et al., 2014; Reuter et al., 2016).

The identification of ADGRL3 provided a disease-relevant target because: (a) it is expressed in brain areas related to attention and activity in human such as the prefrontal cortex, cerebellum, amygdala and temporal lobes (Krain and Castellanos, 2006; Plessen et al., 2006; Arcos-Burgos et al., 2010); (b) ADGRL3-deficient animal models display phenotypes linked to ADHD such as hyperactivity, deficiencies in dopamine and serotonin molecular pathways, but also show a response to MPH treatment in alleviating symptoms (Lange et al., 2012; Wallis et al., 2012; Orsini et al., 2016; van der Voet et al., 2016).

### Autism Spectrum Disorder

Autism spectrum disorder (ASD) is a neuropsychiatric disorder characterized mainly by deficits in social communication and interactions, restricted or repeated actions referred as stereotypic behaviors (Levy et al., 2009). According to different studies the heritability contributes from 54 to 95% of its etiology (Gaugler et al., 2014; Sandin et al., 2014; Colvert et al., 2015). Hence, most studies aiming to elucidate the causes of ASD focused on identifying genetic factors. Interestingly, comorbidity with other neurological diseases often arises when a diagnosis of ASD is given such as the one existing with ADHD. Both disorders present neurological alterations and many of the genes that have been related to their etiology encode synaptic proteins, suggesting that the disorders present dysfunction at the synaptic level (Rowlandson and Smith, 2009; Matson et al., 2010; Guang et al., 2018).

The temporal and frontal lobes are the main brain areas affected in patients with ASD, highlighting the role of the amygdala by its association with aggressive and social behaviors. These areas also contain an important proportion of neurons producing dopamine, a neurotransmitter reinforcing pleasant behaviors through the reward pathway. The mesolimbic pathway which regulates reward processing connects the ventral tegmental area (VTA) of the midbrain and the nucleus accumbens (NAc) of the striatum via white matter tracts (Haber and Knutson, 2010; O’Connell and Hofmann, 2011). Patients with ASD display reduced structural connectivity between the VTA and NAc in the mesolimbic pathway and weaker connectivity in this area translates to more severe social deficits (Supekar et al., 2018). These findings support the hypothesis that patients with ASD find social stimuli less rewarding than their neurotypical peers, which is reflected in their social skills (Sah et al., 2003; Chevallier et al., 2012). Thus, this condition might be related to defects in dopaminergic signaling, a phenotype that is reminiscent of ADHD neuronal deficiencies.

Among the genes whose variants have been related to autism are the genes encoding for synaptic proteins Lphn3, neurexins, neuroligins and SHANK (Wang et al., 2016; Chen et al., 2017; Stessman et al., 2017). It is worth mentioning that both neurexins and the neuroligins form a complex within the synapses which is responsible for recruiting synaptic components such as neurotransmitter receptors and scaffolding proteins to promote an assembly of the synapse as well as its maturation and differentiation (Krueger et al., 2012; Sudhof, 2017). Copy-number variations (CNVs) within NRXN1 have been associated with ASD, however, they are extremely rare and have low penetrance in the general population while NLGN mutations related to ASD exhibited defects in synaptic properties and ASD-like behavioral changes when studied in mouse models (Jamain et al., 2003; Tabuchi et al., 2007; Jaramillo et al., 2014; Todarello et al., 2014). The SHANK family of PDZ domain proteins function as molecular scaffolds at excitatory synapses and through their multiple domains are able to interact with more than 30 synaptic proteins, which confers them an essential role in the formation of synapses (Monteiro and Feng, 2017). Impairments in cognitive function were detected in mice heterozygous for Shank3 with the PDZ domain deleted (Mei et al., 2016). Interestingly, Lphn1 is able to interact with Neurexins to form adhesion complexes (Boucard et al., 2012) while Shank proteins are also able to interact with Lphns PDZ binding domain (Kreienkamp et al., 2000; Tobaben et al., 2000). This network of interaction hints to a common biological pathway underlying the etiology of ASD (Mosca et al., 2017).

### Bipolar Disorder

Bipolar disorder (BD) is a severe chronic mood disorder whose symptoms are episodes ranging from mania, hypomania to severe depression (Vieta and Phillips, 2007). Within the cerebral regions affected in the disease, the hippocampus stands out. In addition to having a critical role in cognitive functions, the hippocampus is also involved in emotion and other functions that are altered in BD such as motivational behaviors and response to stress (Surget et al., 2011; Rive et al., 2013). Several studies have reported hippocampal subfield-level volume reductions in BD, particularly in the right cornu Ammonis 1 (CA1), the granule cell layer (GCL), and the whole hippocampus compared with healthy controls (Haukvik et al., 2015; Han et al., 2019).

Genetic factors play an important role in the disease. The heritability of BD according to twin studies has been estimated to range between 60 and 80%, while lower rates of risk have been found in intergenerational family studies in large population cohorts (Smoller and Finn, 2003; Wray and Gottesman, 2012). Like for many psychiatric disorders, BD presents comorbidity with other mood disorders such as ADHD and/or alcoholism, while displaying pathophysiological defects suggestive of a common etiology based in a monoaminergic imbalance, more specifically alterations of the dopaminergic system, similar to what has been reported for ADHD and substance use disorder, as discussed above (Martinowich et al., 2009; Lydall et al., 2011; Vaughan and Foster, 2013).

Several risk alleles for BD have been identified in genome-wide association studies involving diagnosed patients, among these the following genes encoding Lphn ligands:TENM2, NRXN1, NRXN3 and FLRT2 (Rouillard et al., 2016). Within the chromosomal regions identified as part of the risk loci for BD lies the gene TENM4 encoding teneurin-4 (Craddock and Sklar, 2013; Muhleisen et al., 2014). Interestingly, the teneurin family are known ligands for latrophilins, forming *trans*-synaptic interactions that are suggested to participate in the formation and maintenance of neuronal synapses (Boucard et al., 2014). Although a direct participation of latrophilins in BD has not been reported so far, there could be a pathway associated with the disease in which the Lphns are involved. This hypothesis would be supported by the comorbidity between diseases BD (associated with Ten4) and ADHD (associated with Lphn3) in addition to the alteration of the dopaminergic system observed in latrophilin3-deficient animal models.

### Substance Use Disorder (SUD)

Substance use disorder is an important health problem at a global level, with high economic costs and which is expected to continue to grow over time. This disorder is characterized by a prolonged use of legal or illegal drugs as well as medications, which triggers a loss of self-control (American Psychiatric Association [APA], 2013). Like for other neuropsychiatric diseases, SUD has a strong genetic component. Studies report that the parental background of alcoholism or family history of diagnosis of SUD considerably increase the chances of developing alcohol problems, however, environmental factors also play an important role, which has been shown in studies with twins (Chassin et al., 1991; Agrawal et al., 2010; Huizink et al., 2010).

The areas of the brain that seem to be mostly involved in initial drug reward/saliency are mid-brain dopamine neurons projecting into the prefrontal cortex as well as the dorsal and ventral striatum, these data are supported by imaging studies that show that drug use increases striatal dopamine proportionally to self-reported euphoria (Drevets et al., 2001; Sharma and Brody, 2009; Volkow et al., 2009).

ADHD is a disease that frequently presents comorbidity with SUD. Children diagnosed with ADHD and who were followed up toward adolescence exhibit higher rates of alcohol, tobacco, and psychoactive drug use, as well as greater professional, social and personal impairment than non-ADHD subjects (Molina and Pelham, 2003; Molina et al., 2013; Nogueira et al., 2014). Interestingly, a study investigating whether ADHD risk variants at the ADGRL3 locus interact with clinical, demographic, and environmental variables associated with SUD revealed that the presence of SUD in patients with ADHD can be predicted efficiently, thus identifying ADGRL3 as a risk gene for SUD (Arcos-Burgos and Velez, 2019). In agreement with these results, treatment for ADHD was associated with lower concurrent risk of SUD (Quinn et al., 2017).

### Microcephaly

Genetic alterations during the development of the nervous system are one of the main causes that lead to malformations of cortical development (MCP). Microcephaly is a type of MCP that is characterized by a reduction in the circumference of the head of a human, where infectious, environmental and genetic factors are considered as the causative agents in this condition (Parrini et al., 2016). During the development of the cerebral cortex, cell proliferation, neuronal migration or postmigrational cortical and connectivity are key stages for a successful development and any defect in the regulation of these cellular processes can lead to different types of MCP. Particularly in microcephaly there is a deregulation in DNA replication that leads to the decrease of cell proliferation, and therefore to the aforementioned phenotype (Barkovich et al., 2012; Kalogeropoulou et al., 2019). It has been reported that patients with defects in a single gene display comorbidity between microcephaly and other MCPs, such as lissencephaly (which is associated with deficiencies in neural migration) and agenesis of the corpus callosum (ACC) and more recently with rhombencephalosynapsis (RES)(Parrini et al., 2016). RES is an extremely rare malformation in which there is no anatomical differentiation of the cerebral hemispheres (Aldinger et al., 2018). A new variant in the LPHN2 gene was detected in a sample from a human fetus which presented severe microcephaly, severely reduced sulcation and RES. This nonsense variant resulted in the change of a leucine to a histidine at position 1262 of its intracellular domain, which affected its functionality in the mobilization of calcium through its coupling to G proteins and the organization of the cytoskeleton, promoting an increase in the cell adhesion and decrease in cell migration, processes which are of crucial importance in cortical development (Vezain et al., 2018). These findings highlight the regulatory capacity of Lphn2 in determining the etiology of neurodevelopmental disorders.

### Schizophrenia

Schizophrenia (SCZ) is a neurological disorder affecting approximately 1% in the world population. Patients with this disorder usually present psychotic symptoms (hallucinations), social withdrawal and deficits in attention and working memory. Schizophrenia usually displays late adolescence onset or early adulthood onset and is considered multifactorial (Kellendonk et al., 2009). However, genetic factors contribute approximately 60–80% in its etiology. At the molecular level, alterations in the synthesis and release of dopamine were detected in the striatum and in the dorsolateral prefrontal cortex of affected individuals (Howes et al., 2012; Conio et al., 2019). Recently, a single nucleotide variation in an intronic sequence of the ADGRL2 gene was reported in patients diagnosed with schizophrenia who were prescribed clozapine, an antipsychotic usually recommended for the treatment of schizophrenia (Legge et al., 2018). Deletions or polymorphisms in the genes that encode Lphn ligands neurexins and teneurins, have also been associated with this condition (Kirov et al., 2008; Gauthier et al., 2011; Ivorra et al., 2014). Notably, association studies with SCZ identified a single nucleotide modification in NRXN1 gene which resulted in poor synaptic differentiation and loss of interaction with its canonical ligand, neuroligin, in neuronal co-cultures; in addition, variants located in the YD repeat domain of teneurin-4 were also identified in samples from SCZ patients some of which presented a comorbidity with bipolar disorder (Yamada et al., 2004; Kirov et al., 2008; Gauthier et al., 2011; Xue et al., 2018). Although the contribution of latrophilins to the etiology of schizophrenia is unknown, their role in synapse formation and their association with the regulation of dopaminergic signaling constitute key features that warrant a closer look at the pathophysiological functions of these molecules for this psychiatric disorder.

### Epilepsy

Epilepsy is a disorder that is characterized by the presence of seizures presumably because of an alteration in the balance between excitatory and inhibitory impulses in the brain. This condition usually presents comorbidity with other disorders such as depression, SCZ and MCP. There are different types of epilepsy according to the type of convulsion, the affected brain area, age of the patient, and etiological factors. Its heritability is high but there are also sporadic cases where its condition is related to environmental factors (Stafstrom and Carmant, 2015; Hauser et al., 2018). Mutations in genes that code for sodium (SCN1A) and potassium (KCNA2) channels and N-methyl-D-aspartate (NMDA) receptors have been highly associated with their condition (Perucca and Perucca, 2019). However, there is a significant interest in the study of new variants related to epilepsy among them neurexins and contactin-6, ligands that interact with latrophilins. A female infant with Early infantile epileptic encephalopathy presented variations of a single nucleotide in the NRXN1 and 2 genes generating a missense mutation in the corresponding proteins (Rochtus et al., 2019). In another study of patients with generalized epilepsies (IGEs) of European ancestry, exon disrupting deletions were reported in the promoter region of NRXN1 (Moller et al., 2013). On the other hand, the relationship between epilepsy and variations in the contactin-6 gene (CNTN6) remains scarce, but a deletion of exons 21 and 22 has been highly associated with the presence of schizophrenia and seizures (which is the hallmark symptom of epilepsy) (Juan-Perez et al., 2018). Thus, of the two latrophilin ligands, neurexins associations with epilepsy retain the most interest. As for latrophilins, the evidences are scarce and can be summed up to a study in patients diagnosed with partial epilepsy of European ancestry which reported five variants in different intronic regions of the ADGRL3 gene; however, its relationship with the disease did not reach significance (Kasperaviciute et al., 2010). This result does not nullify the possibility of its relationship with the disease, because due to its multifactorial etiology, ADGRL3 could be related to other types of epilepsy. Lphn3 role in modulating the formation of specific excitatory synaptic contacts (Sando et al., 2019) suggest this molecule as a potential epilepsy risk factor giving that some of its variants could lead to an imbalance at the excitatory level that generates symptoms related with this disorder.

## Concluding Remarks and Hypothesis

Latrophilins are bound to affect proper neuronal functions given their conserved expression in this cell type, from mechanosensation in *Drosophila*, to pharyngeal pumping in *C. elegans* or learning in *M. musculus*. While some specific roles of latrophilins across organisms may vary, the underlying basic mechanisms that rely on their domain structure are likely conserved. Their adhesion function in mammals requires heterophilic interactions with teneurins, but this binding profile has not been replicated in invertebrate animal models. Latrophilins in these organisms are likely to form complexes with teneurins given that (a) the Lectin-like domain of latrophilin that binds teneurins in mammals is conserved in invertebrates, (b) the expression of both adhesion molecules overlaps with one another in certain tissues or are directly adjacent.

Latrophilins’ association with numerous psychiatric disorders hints to their importance in the modulation of cognitive functions in humans. Animal models deficient in latrophilin-3 orthologs display behavioral phenotypes that relate to the human condition of ADHD and respond to clinically relevant medication, thus suggesting an interspecies role of this receptor in regulating dopaminergic pathways. However, more needs to be done to understand the underlying biological role of latrophilins. Our theory is that latrophilins, by transducing adhesion events into G protein-dependent and G protein-independent cell signaling cascades are relevant for neuronal development and brain functions. Furthermore, latrophilins mediate synaptogenesis and therefore the plastic behavior of the nervous system. We propose that latrophilins can act both in *cis* and *trans* configurations with their ligands to produce signaling complexes that can elicit configuration-dependent signaling schemes. Despite significant progress in various models, more work is required to identify the specific contexts in which these receptors function.

## Data Availability

All datasets generated for this study are included in the manuscript and/or the Supplementary Files.

## Author Contributions

AM-S, MA-Z, and AB reviewed the literature. AM-S, MA-Z, FM, and AB drafted and designed the manuscript. PU-S electronically drew and assembled all figures. AB, AM-S, MA-Z, PU-S, and FM provided the concept for the figures and discussed figure details. AM-S, MA-Z, and AB conducted and analyzed the alignment data. DH-G, FM, and AB provided experimental data and analysis of microscopy images. All authors provided intellectual inputs, revised, and approved the final version of the manuscript.

## Conflict of Interest Statement

The authors declare that the research was conducted in the absence of any commercial or financial relationships that could be construed as a potential conflict of interest.
