# Supplementary figures and images for: Latrophilins: A Neuro-Centric View of an Evolutionary Conserved Adhesion G Protein-Coupled Receptor Subfamily
Source: Front Neurosci. 2019 Jul 9;13:700. doi: 10.3389/fnins.2019.00700 (PMC6629964; doi:10.3389/fnins.2019.00700)

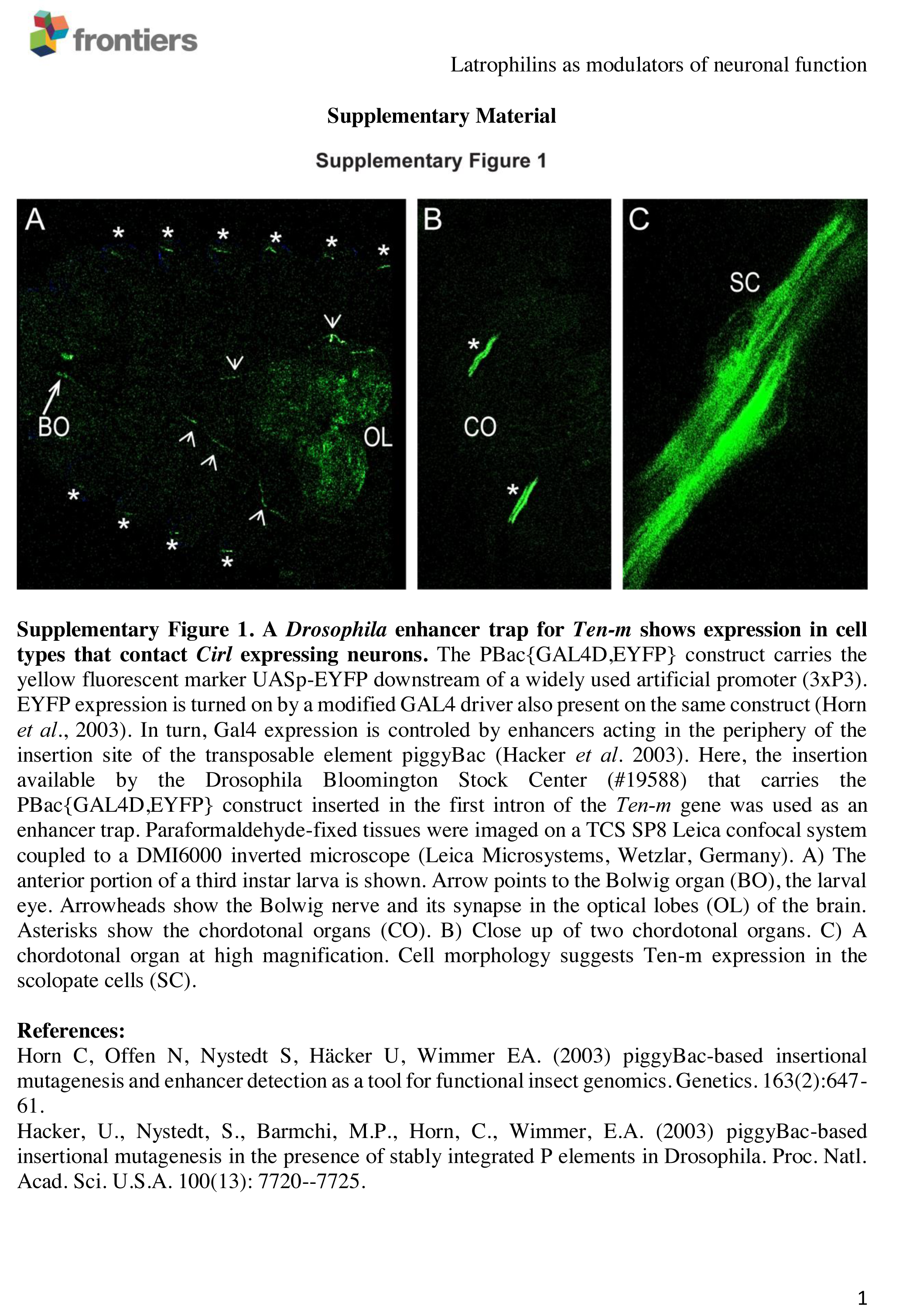

Supplement: Supplementary file 1 [file Image_1.TIF]
